# Factors Associated with Platelet Activation-Recent Pharmaceutical Approaches

**DOI:** 10.3390/ijms23063301

**Published:** 2022-03-18

**Authors:** Panagiotis Theofilis, Marios Sagris, Evangelos Oikonomou, Alexios S. Antonopoulos, Konstantinos Tsioufis, Dimitris Tousoulis

**Affiliations:** 1 Cardiology Department, “Hippokration” General Hospital, University of Athens Medical School, 11527 Athens, Greece; panos.theofilis@hotmail.com (P.T.); masagris1919@gmail.com (M.S.); boikono@gmail.com (E.O.); antonopoulosal@yahoo.gr (A.S.A.); kptsioufis@gmail.com (K.T.); 2 Cardiology Department, “Sotiria” Chest Diseases Hospital, University of Athens Medical School, 11527 Athens, Greece

**Keywords:** platelet activation, inflammation, endothelial dysfunction, miR, P2Y12

## Abstract

Platelets are at the forefront of human health and disease following the advances in their research presented in past decades. Platelet activation, their most crucial function, although beneficial in the case of vascular injury, may represent the initial step for thrombotic complications characterizing various pathologic states, primarily atherosclerotic cardiovascular diseases. In this review, we initially summarize the structural and functional characteristics of platelets. Next, we focus on the process of platelet activation and its associated factors, indicating the potential molecular mechanisms involving inflammation, endothelial dysfunction, and miRs. Finally, an overview of the available antiplatelet agents is being portrayed, together with agents possessing off-set platelet-inhibitory actions, while an extensive presentation of drugs under investigation is being given.

## 1. Introduction

Recent advances in the field of platelet research have provided a better understanding of their biology and function. This essential knowledge has been incorporated in clinical scenarios, leading to further elucidation of their role in the pathogenesis of various disease states. Ultimately, targeting platelets has become a cornerstone of the therapeutic approach, especially in atherosclerotic cardiovascular diseases, which are characterized by arterial thrombosis. However, novel concepts have also linked platelets with the initiation or augmentation of an immune response through their activation. In this review, we provide an overview of the available evidence concerning the activation of platelets, together with the latest data on their pharmacologic inhibition.

## 2. Overview of Platelet Biology and Main Functions

### 2.1. Platelet Structure and Formation

Platelets are anucleate, small (2–4 μm in diameter) blood cells with quintessential roles in human health, as well as in the development various pathologic processes. Their lifespan ranges from 7 to 10 days, and they are ultimately cleared by the spleen and liver. As far as their formation is concerned, which takes place in the bone marrow, hematopoietic stem cell differentiation into megakaryocytes (MK) and proplatelets represents the initial step.

Thrombopoietin, a glycoprotein produced in the liver, plays a crucial role in this process as it is able to influence platelet production through negative feedback regulation. Thrombopoietin receptors are present on the surface of megakaryocytes and platelets. Binding of thrombopoietin to the megakaryocyte receptor leads to the maturation of megakaryocytes, while thrombopoietin degradation depends on its binding on the platelet receptor [1]. Thus, platelet and megakaryocyte counts regulate the action of thrombopoietin. This is especially evident in states of thrombocytopenia where thrombopoietin stimulates its receptor on the surface of platelets, the myeloproliferative leukemia protein (MLP), inducing its dimerization and activation of janus kinase 2 (JAK2). Ultimately, JAK2 phosphorylates MLP, which in turn leads to the recruitment of signal transducers and activators of transcription (STATs), phosphoinositide-3-kinase (PI3K), and mitogen-activated protein kinases (MAPKs) [2,3]. Additionally, inflammatory stimuli such as interleukin (IL)-6 may also enhance thrombopoiesis by increasing the levels of thrombopoietin, thus inducing reactive thrombopoiesis [4]. Other factors have also been implicated, including insulin-like growth factor 1 (IGF1) and the activated form of tyrosine-tRNA ligase, C-C motif chemokine 5 (CCL5), and IL-1α, among others [5,6]. Several transcription factors along with their cofactors influence the maturation of MK at the intercellular level [7]. Following the differentiation and maturation of MK, they interact with endothelial cells lining the bone marrow vasculature and then protrude and elongate, as proplatelets, with the aid of specialized podosomes [8]. These special structures may be responsible for the breach in the endothelial barrier [8,9]. Consequently, podosomes form actin-rich anchors extending to the luminal surface of endothelial cells [10]. Proplatelet remodeling, fragmentation, and release represents the final step in this cascade, with the available evidence pointing towards the lung microcirculation as the primary site of this process due to a favorable hemodynamic profile [11,12].

### 2.2. Platelet Functions

Platelets orchestrate multiple processes, among which the most well-characterized is hemostasis and thrombosis [13]. This function has been believed to be the most prominent platelet function until recent years [14]. In order to perform this role, platelets flow proximal to the vessel wall with the aid of the vascular shear forces, so that a quick response to an injury may be secured [13]. In cases of vascular injury, the initial step consists of the adhesion to the subendothelial matrix through the binding of glycoprotein (GP)Ib/V/IX complex to von Willebrand factor (VWF) and the binding of GPVI and α2β1 to extracellular collagen. Next, more potent adhesive bonds are established and the initial clot is being formed. The activation of the integrin receptor GPIIb/IIIa, initiated by various agonists or through inside-out signaling, and the subsequent binding of the symmetrical fibrinogen, is essential to the attraction of surrounding platelet (platelet-platelet interactions) [15]. A feedback loop persists, mediated by cyclooxygenase-1, 12-lipooxygenase, and the secreted granule contents, leading to the subsequent activation of the remaining platelets. Ultimately, platelet aggregation leads to the formation of the hemostatic plug or thrombus in cases of vascular injury or atherosclerotis, respectively. Other than their cemented role in hemostasis, platelets are believed to contribute towards the immunity by regulating inflammatory responses [16,17]. In this regard, platelet toll-like receptors (TLRs), which are pattern recognition receptors, are beginning to be investigated [18]. Lastly, the role of platelet-derived microparticles in thromboinflammatory diseases has also been described [19].

## 3. Molecular Mechanisms of Platelet Activation

**Figure 1:**
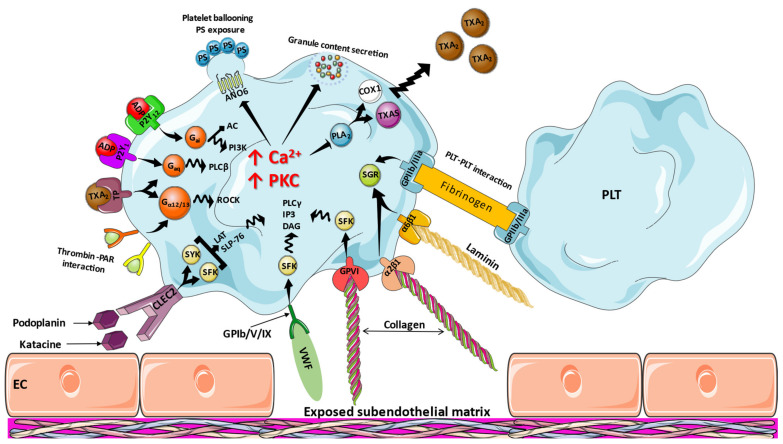
Mechanisms of platelet activation. AC: adenylyl cyclase, ADP: adenosine diphosphate, ANO6: anoctamin 6, CLEC2: C-type lectin-like receptor 2, COX1: cyclooxygenase 1, DAG: 1,2-diacyl-glycerol, EC: endothelial cell, GP: glycoprotein, LAT: linker for activation of T cells, PAR: protease activater receptor, PI3K: phosphoinositide 3-kinases, PLA_2_: phospholipase A_2_, PLC: phospholipase, PLT: platelet, PS: phosphatidylserine, ROCK: Rho-associated protein kinase, SFK: SRC-family kinase, SGR: small G-protein regulator, SLP-76: lymphocyte cytosolic protein 2, TP: thromboxane receptor, TXA_2_: thromboxane, TXAS: thromboxane-A synthase, VWF: von Willebrand factor.

The process of platelet activation is multistep, involving numerous factors (Figure 1). In the case of vascular injury, adhesion receptors on platelet surface, such as the integrins α6β1, α2β1, GPIIb/IIIa, and the GPIb/V/IX complex bind to laminin, collagen, fibrinogen, and VWF, respectively, at the presence of small G-protein regulators (SGRs), SRC-family kinases (SFKs), and serine/threonine-protein kinases (STKs) [20,21,22]. As a result, a change in platelet shape occurs. C-type lectin-like receptor 2 (CLEC2) may also be activated through its endogenous ligand podoplanin, as well as by the newly identified katacine [23], clustering on platelet surface and inducing protein tyrosine kinase pathways (involving mostly SYK but also SFK) and adapter proteins (Linker for activation of T cells, Lymphocyte cytosolic protein 2). Subsequently, activation of phospholipase Cγ (PLCγ), IP3, and 1,2-diacyl-glycerol (DAG) and Ca^2+^ release into the cytoplasm [24]. GPVI, the most potent platelet activator during adhesion through collagen exposure, acts through a similar pathway but involves only SFKs [25]. Additionally, the release of platelet agonists such as adenosine diphosphate (ADP) and thromboxane A2 activate the P2Y_12_, P2Y_1_, and thromboxane receptor [26]. These events are particularly important since P2Y_12_, through the action of G protein Gαi, inhibits adenylyl cyclase, an enzyme responsible for the conversion of adenosine triphosphate to the antiaggregant cyclic adenosine monophosphate (cAMP), and stimulates phosphoinositide 3-kinases (PI3Ks), while P2Y_1_ and thromboxane receptor signal via Gαq proteins, stimulating phospholipase Cβ, thus also inducing release of calcium into the cytoplasm and protein kinase C activation [27]. The action of the thromboxane receptor extends to the stimulation of G_α12/13_ proteins, which are responsible for triggering Rho-associated protein kinase (ROCK) activation [28]. ROCK activation is also seen with Thrombin-mediated protease-activated receptors (PAR) 1 and 4, which are also G protein-coupled receptors [29]. Consequently, Ca^2+^ promotes phospholipase A_2_ activation, which cleaves arachidonic acid. The released arachidonic acid is substrate for cyclooxygenase-1 and next thromboxane synthase, ultimately leading to the formation of thromboxane A2. Moreover, through increased Ca^2+^ and DAG, secretion of granule contents takes place [30], containing ADP and VWF, among others [31]. Platelet ballooning and phosphatidylserine exposure ensues in response to high calcium concentrations, through the anoctamin 6 ion channel, leading to increased procoagulant ability and intracellular protein degradation [32].

### 3.1. Inflammation

Inflammatory stimuli, increasingly being found in various disease states [33,34,35], have been implicated in platelet activation and subsequent thrombosis (Table 1) [36,37]. Among the main inflammatory mediators, pro-inflammatory cytokines may augment the risk of thrombosis through platelet activation, since platelets possess receptors for ILs which are stimulated in situations of inflammation. Platelet IL-1 receptor has been the most studied [38], while the presence of platelet receptors for IL-6 and IL-8 was previously described [39,40]. Confirmatory to this hypothesis, platelet hyper-reactivity was observed when whole blood from healthy individuals was exposed to IL-1β, IL-6, IL-8, and IL-12 [41,42]. Tumor necrosis factor-alpha (TNF-α) constitutes another molecule of potential importance towards platelet activation. It was recently proposed that TNF-α may be the most prominent cytokine inducing platelet hyper-reactivity, even at the level of bone marrow, by increasing the megakaryocyte precursors and the megakaryocyte ploidy status, among others in old (>18 months) C57BL/6J mice [43]. Other inflammatory pathways were also genetically altered (IL-1β, IL-6, TNF receptor-1 and -2), together with metabolic pathways and mitochondrial function. These findings were replicated when young wild type C57BL/6J mice were given recombinant TNF-α. Such observations were not noticed when IL-1β was administered. The presence of functional TNF-α receptors consist of a fundamental prerequisite for the induction of platelet hyper-reactivity, however.

Moving to the platelet-derived nucleotide-binding oligomerization domain, leucine-rich repeat–containing receptor (NLR) family pyrin domain-containing 3 (NLRP3) inflammasome, several reports have stated its importance in platelet activation. Its action may be elicited either in the presence of an activated platelet IL-1 receptor or without the need of an initial primer [44,45]. The platelet-activating effect of NLRP3 inflammasome may be mediated by the platelet bruton’s tyrosine kinase (BTK), as shown by Vogel et al., who detected platelet activation and thrombus formation to a lesser extent after administration of the BTK inhibitor ibrutinib in sickle cell mice [46]. BTK is also responsible for GPIb and GPVI signaling, thus constituting an attractive treatment target [47].

Platelet-neutrophil interactions in the setting of inflammation have also garnered scientific interest. Platelet binding of their surface protein P-selectin with its ligand P-Selectin glycoprotein ligand-1 on the neutrophil’s surface consists of the main mechanism of their activation. The interaction between platelet GPIb and neutrophil integrin αMβ2 or the binding of platelet GPIIbIIIa with neutrophil integrin αMβ2 constitute alternative pathways [48]. Other molecules which may be implicated in the process of platelet activation are the PAR-4 through the secretion of dense granule contents [49], and thrombospondin-1 which is proteolyzed by neutrophils into a molecule with potent platelet-activating properties [50]. Moreover, the abundance of cytokines in inflammatory conditions lead to the overexpression of adhesion molecules which bind with platelets, augmenting platelet activation [37]. Neutrophils may also induce platelet activation via novel, incompletely understood pathways involving human neutrophil peptides [51,52], heparin binding protein [53], calprotectin [54,55], cathelicidins [56], and neutrophil cathepsin G [57]. The stimulation of platelets by neutrophil extracellular vesicles and the subsequent increase in thromboxane A2 synthesis may be an additional mediator of platelet activation [58]. Therefore, the interaction between platelets and neutrophils leads to further activation of the inflammatory and thrombotic cascades, in a process termed thromboinflammation.

The role of recently discovered neutrophil extracellular traps (NETs) deserves an honorable mention since they have been implicated in thromboinflammatory diseases such as atherosclerosis [59]. Based on their structure, consisting of DNA, histones, and granular components, NETs are believed to be thrombosis-inducers through direct contact coagulation or platelet activation in a histone-dependent manner, by recruitment of TLRs [60,61]. High-mobility group box 1 is among the known TLR4 stimulants which has been implicated in NET-induced thrombosis. The presence of NETs has been associated with the presence of thrombi in coronary and cerebral circulation [62,63], indicating their importance in thromboinflammation.

As far as platelet TLRs are concerned, preliminary evidence suggests a pro-thrombotic role of platelet TLR2/1, mediated by the action of nuclear factor-kappaB (NF-κB) or adenosine diphosphate or triphosphate together with thromboxane A2 [64,65,66]. Less well understood is the role of platelets TLR4, TLR2/6, TLR3, and TLR7 which appear to be incapable of producing a potent platelet-activating effect [67,68,69,70,71,72]. More research is required in the field of platelet TLRs, however, as their role may be pro-inflammatory rather than pro-thrombotic.

### 3.2. Endothelial Dysfunction

Disrupted endothelial cell homeostasis is a major factor influencing platelet activation (Table 1), which is among the numerous functions regulated by the vascular endothelium. Several regulators have been described in this anti-platelet effect. First and foremost, nitric oxide (NO), a well-known vasodilating substance, may attenuate platelet activation through the activation of soluble guanylate cyclase. As a result, increased production of intracellular cyclic guanosine monophosphate (cGMP) activates protein kinase G (PKG), which phosphorylates Ca-releasing channels and hence results in lower intracellular calcium levels, which are critical for the initiation of platelet activation [73]. In the setting of a dysfunctional endothelial layer, an impaired NO bioavailability is noted owing to diminished production, enhanced degradation, and endothelial NO synthase uncoupling. Ultimately, the formed peroxynitrite may aid platelet activation [74].

Prostacyclin (PGI_2_) is another molecule stemming from the endothelial cells, with inhibitory actions towards platelet activation. After its binding with the prostacyclin receptor on the surface of platelets, the adenylate cyclase-driven cAMP formation leads to the phosphorylation of the IP3 receptor, resulting again in a lowering of the intracellular calcium, thus halting platelet activation [75]. It should be noted that the action of PGI_2_ is opposed by thromboxane A2 [76]. Ectonucleotidases are also able to exert an anti-platelet effect. Among the most well-characterized is CD39, which hydrolyzes the platelet-activating ADP to adenosine monophosphate (AMP), and CD73, which transforms AMP to adenosine that, by binding to its platelet receptor A2A, activates Gαs, adenylyl cyclase with again increased production of cAMP, thus blocking platelet activation [77]. Thrombomodulin (TM), a transmembrane glycoprotein expressed primarily on the surface of endothelial cells (but also on neutrophils, macrophages, monocytes, and dendritic cells), is among the main inhibitors of platelet activation [78]. Its role revolves around binding thrombin, thus reducing its ability to convert fibrinogen into fibrin and activate platelets [78].

VWF is also implicated in platelet activation. It is mainly produced in the endoplasmic reticulum of endothelial cells and stored in the Weibel-Palade bodies as well as in platelet α-granules [79,80]. Although constitutive VWF secretion occurs physiologically, agonist-induced endothelial cell activation leads secretion of ultralarge VWF molecules from Weibel Palade bodies. Consequently, they exert their platelet-activating action by bonding of their A1 domain, which is activated in areas of vascular injury or very high shear stress, with platelets via their receptor GPIb/V/IX [81]. However, under physiologic conditions, ultralarge VWF multimeres are cleaved into smaller forms primarily by A disintegrin-like and metalloprotease with thrombospondin type-1 repeats-13 (ADAMTS13) at the Tyr1605-Met1606 site of the A2 domain [82]. These small VWF molecules are present in the circulation under physiologic conditions. Last but not least, the role of the endothelial glycocalyx (eGC) ought to be stressed. As the 0.5–5μm thick cover of endothelial cells at their luminal surface, it is composed of proteoglycans, glycolipids, glycoproteins, and glycosaminoglycans [83]. Under homeostatic conditions, there is a fine balance between eGC components being shed and formed, leading to the stabilization of this barrier.

In the setting of a dysfunctional endothelial layer, the above-mentioned beneficial antiplatelet effects are abolished. An impaired NO bioavailability is being noted owing to diminished production, enhanced degradation, and endothelial NO synthase uncoupling in cases of endothelial activation by oxidative stress and inflammation. Ultimately, the formed peroxynitrite may aid platelet activation [74]. Similarly, endothelial dysfunction may aid the thrombotic process through the disruption of the PGI_2_-Thromboxane A2 balance in favor of thromboxane A2 [76]. Lower levels of prostacyclin synthase, the catalyst of PGI_2_ synthesis, were found in the subcutaneous arteries of individuals with diabetes mellitus, a condition characterized by profound endothelial dysfunction, compared to matched controls, possibly leading to impaired transformation of prostaglandin E_1_ to PGI_2_ [84]. The production of TM is also diminished under conditions of endothelial dysfunction, with a subsequent lack of its anti-platelet effect [78]. Concerning ectonucleotidases, a downregulated expression of CD39 has been noted in conditions associated with endothelial dysfunction, such as experimental models of arterial hypertension, paired with diminished arterial nucleotidase activity [85]. The loss of ectonucleotidase activity was also paired with ATP-, AMP, and adenosine-degrading enzymes in a model of ischemic heart disease [86]. Lastly, increased eGC degradation and thinning by sheddases in pathologic states [87,88,89] promotes a prothrombotic state due to the facilitated binding of leukocytes and platelets on the receptors of adhesion molecules and VWF released by endothelial cells [90,91,92].

### 3.3. MiRs

MiRs have been implicated in human diseases pathophysiology, diagnosis, and treatment [93,94,95,96]. Platelet activation is among the numerous function that are being regulated by miRs (Table 1). Several miRs are expressed in high amounts by platelets, including miR-223, miR-126, miR-197, miR-24, and miR-21 [97]. Focusing on miR-126, its role in thrombosis-driven diseases such as atherosclerosis has been thoroughly studied, and its influence on platelet activation may be among its important regulatory functions [98]. As a result, treatment with antiplatelet agents may lower miR-126 levels, while the downregulated expression of this miR in the setting of diabetes mellitus might be another factor that promotes thrombosis in this pathologic state [99]. Although platelet miR-126 was not associated with platelet activity indices in patients with an ST-elevation myocardial infarction, it correlated with plasma cardiac troponin I [100]. Moving to miR-223, one of the most abundant platelet-derived miRs [101], its association with the P2Y_12_ receptor and secreted modular calcium-binding protein 1 regulation has been described [101,102]. Even though Leierseder et al. noted no effect of miR-223 on platelet function, it could be argued that the dosage of platelet agonist may have overshadowed the effect of miR-223 [103]. The levels of two miRs mentioned above were significantly correlated with P-selectin and platelet factor 4 together with significant positive correlations with the vasodilator-stimulated phosphoprotein phosphorylation assay [104]. As far as miR-197, miR-24, and miR-21 are concerned, there is no reliable evidence regarding their role in platelet activation. MiR-320c and miR-181 have also been recently investigated as they may be implicated in the downregulation of Ras-related Protein 1, which is secreted in large amounts following agonist-induced platelet activation [105,106]. Decreased platelet and MK miR-26b was associated with enhanced platelet activation, evidenced by increased expression of P-selectin, in septic conditions [107]. Augmented platelet reactivity could also be attributed to miR-15b-5p, miR-93, and miR-126 in a study of post-acute coronary syndrome patients [108]. Novel miRs associated with platelet activation in patients with stable coronary artery disease were reported by Pedersen et al., that included miR-93-5p, miR-150-5p, miR-423-3p, and miR-1180-3p, as well as miR-126-3p [109]. MiR-150-5p may be involved in the regulation of actin cytoskeleton and calcium homeostasis [110]. MiR-204-5p was also associated with platelet aggregation, potentially regulating genes involved in actin cytoskeleton and P2Y_12_ pathways [110].

Platelet miRs have also been investigated in 22 patients with essential thrombocythemia [111]. The investigators noted that miR-9 and miR-490 were upregulated in the study group compared to healthy volunteers, while miR-10a, miR-28, miR-126, miR-155, miR-221, miR-222, miR-223, and miR-43 were downregulated [111]. As far as platelet activation is concerned, the expression of miR-126 was correlated with the degree of platelet aggregation induced by arachidonic acid (AA) and thrombin-receptor-activating-peptide (TRAP) [111]. Additionally, miR-9 and miR-490 were inversely correlated with the percentage of fibrinogen-positive, CD63-positive, and P-selectin-positive platelets when stimulated by TRAP [111].

Platelet reactivity on antiplatelet therapy, another crucial matter in patients with atherosclerotic cardiovascular diseases, may be closely linked to miRs [112]. Antiplatelet therapy with increasing doses of aspirin and prasugrel has resulted in decreases of plasma miR-223, miR-191, miR-150, miR-126, in treatment-naïve healthy subjects and in individuals with symptomatic carotid stenosis on aspirin with the addition of dipyridamole or clopidogrel [113]. Plasma miR-223 was also decreased after treatment with clopidogrel and ticagrelor in a recent study on healthy volunteers [114]. More potent P2Y_12_ inhibitors may further lower plasma miR-223 levels, together with miR-150 and miR-126 [115]. It should be noted that contrasting results have also been reported [116]. Interestingly, when platelet miR-223 levels were low, the response to P2Y_12_ inhibitors was diminished [117], and it could be argued that decreased miR-223 could be a predictor of low response to antiplatelets for patients with non-ST-elevation acute coronary syndrome [118]. Indeed, levels of platelet miR-223 were significantly increased in the platelets of patients with an acute coronary syndrome characterized as high responders [119]. An interesting study was conducted by Liu et al. who initially examined the expression of platelet miRs in healthy volunteers characterized as having very high and very low platelet reactivity based on the results of thromboelastography [120]. After identifying lower levels of miR-223 and miR-126 and higher levels of miR-150 in the very high platelet reactivity group, they proceeded to the validation of those results in a cohort of patients with an acute coronary syndrome [120]. Patients with high on-treatment platelet reactivity under dual antiplatelet therapy (DAPT) were characterized by decreased miR-223 and miR-126, with increased miR-50 [120]. In a systematic review conducted by Pedersen et al. concerning the role of miRs in platelet function, miR-223 levels were inversely correlated with platelet reactivity in most studies of patients with an acute coronary syndrome [121]. Conflicting results were reported for miR-126, which was positively correlated in most studies, however [121]. Findings concerning miR-150 were suggestive of a poor association between this miR and platelet function [121]. As most of the mentioned miRs were only assessed in single studies, safe conclusions cannot be drawn regarding their relationship with platelet reactivity [121].

## 4. Therapeutic Approaches

Several therapeutic options are available for the inhibition of platelet activation, with aspirin and P2Y_12_ inhibitors being the mainstay. However, other antiplatelet agents may be situationally important, while the offset effects of other drug categories, such as anti-inflammatory medications, may involve the attenuation of platelet activation. 

### 4.1. Antiplatelet Drugs

#### 4.1.1. Aspirin

Antiplatelet therapies have been extensively studied during the past decades as they remain the cornerstone of treatment of atherosclerotic cardiovascular diseases. Beginning with low-dose aspirin, its ability to selectively and irreversibly inhibit cyclooxygenase-1 has led to its widespread use in primary and secondary prevention of adverse cardiovascular events. Numerous meta-analyses have assessed aspirin’s efficacy in this regard, with remarkable efficacy in reducing cardiovascular events, especially in the context of secondary prevention. As far as primary prevention is concerned, aspirin treatment should be tailored to each individual according to the latest evidence not supporting its universal use [122]. In patients with diabetes mellitus, its use may be warranted according to the risk factor profile [123,124].

#### 4.1.2. P2Y_12_ Inhibitors

P2Y_12_ inhibitors further inhibit platelet activation and have also been used, either as monotherapy or in combination with aspirin, in the prevention of adverse cardiovascular events. Clopidogrel, the most well-studied representative of this class, is administered as a prodrug which is converted by cytochrome P (CYP) 2C19 to the active metabolite, thus being susceptible to competition for the enzyme [125]. Compared to aspirin alone, DAPT may be efficacious in acute minor stroke or transient ischemic attacks [126], established cardiovascular disease [127], and in patients with diabetes mellitus [128], at the cost of increased bleeding rates. It should be noted, however, that in the presence of a CYP2C19 loss-of-function allele, patients treated with clopidogrel for stable coronary artery disease or after percutaneous revascularization for an acute coronary syndrome may be facing an increased risk of major adverse cardiovascular events [129,130,131]. P2Y_12_ gene polymorphisms might also increase the incidence of atherosclerotic events in patients treated with clopidogrel [132]. No such associations were noted concerning the concomitant use of proton pump inhibitors, however [133].

With the development of novel, more potent P2Y_12_ inhibitors, scientific research has focused on the comparison of the efficacy and safety of these agents compared to clopidogrel in the context of coronary syndromes. Concerning loading doses prior to elective percutaneous coronary intervention, no differences in adverse events or bleeding rates were noted [134]. However, prasugrel and ticagrelor have a favorable efficacy profile with an increased risk of bleeding in patients after an acute coronary syndrome, while ticagrelor may also affect mortality [135]. This could potentially be explained by the reversible inhibition provoked by ticagrelor compared to the irreversible inhibition of prasugrel or clopidogrel, which might be of importance in cases of severe bleeding. As far as special patient populations are concerned, patients with chronic kidney disease may benefit from the use of prasugrel [136], while the use of the novel, potent P2Y_12_ inhibitors may reduce the risk of ischemic events at the expense of excess bleeding in the elderly [137]. Intriguingly, a recent meta-analysis demonstrated the key role of the CYP2C19 loss-of-function, as it was a critical moderator of the difference in efficacy between clopidogrel and ticagrelor/prasugrel [138]. Therefore, as stated by Galli et al. in their meta-analysis, a genetically-guided choice of P2Y_12_ inhibitor might provide the ideal combination of safety and efficacy [139]. It should be noted that the de-escalation of anti-platelet therapy from prasugrel or ticagrelor to clopidogrel might be associated with a net clinical benefit in patients treated with percutaneous coronary intervention following an acute coronary syndrome [140].

Despite the existence of potent P2Y_12_ inhibitors for the management of coronary artery disease, a novel candidate was recently proposed by the name of selatogrel (ACT-246475). As a reversible, selective, high-potency P2Y_12_ inhibitor, its administration in a rat model of carotid artery thrombosis led to similar thrombus resolution raters compared to ticagrelor, with a wider therapeutic window and presumed improved safety profile evidenced by reduced blood loss [141]. A possible explanation of this favorable safety profile could be the lack of influence on the stability of hemostatic seals in comparison to other P2Y_12_ inhibitors [142]. In a clinical scenario, the use of subcutaneous selatogrel in 345 patients with stable coronary artery disease on baseline oral antiplatelets was associated with rapid onset, long-lasting, and dose-dependent P2Y_12_ inhibition, significantly increased compared to placebo [143]. At the same time, inhibition of platelet aggregation was noted via light transmission aggregometry [143]. Concerning side effects, 7% of the patients experienced transient dyspnea [143]. This impressive antiplatelet effect was further documented in 47 patients with an acute myocardial infarction who received subcutaneous selatogrel 8 mg or 16 mg followed by ticagrelor administration, where seratogrel induced P2Y_12_ inhibition as early as 15 minutes post-administration, with a high response rate at both dosing regimens [144]. It appears that the combination of selatogrel with ticagrelor might produce the most pronounced platelet inhibitory activity, while a time gap between selatogrel administration and prasugrel/clopidogrel intake needs to be implemented so that the pharmacodynamic effects of the latter remains intact [145]. Selatogrel may also possess a thrombolytic role as recently proposed by Cresence et al., who noted dissolution of the occlusive part of preformed thrombi, with the remaining part potentially serving as hemostatic seal at the site of vessel injury [146]. Future randomized controlled trials with major clinical endpoints may shed more light in the potential of this novel molecule in acute atherothrombotic scenarios.

#### 4.1.3. Glycoprotein IIb/IIIa Inhibitors

GPIIb/IIIa are among the crucial integrins involved in platelet activation. The inhibitors of these molecules, administered intravenously, have been widely used in randomized trials of percutaneous coronary interventions. Especially in high-risk individuals with an acute coronary syndrome, the use of GP inhibitors resulted in lower risk of incident myocardial infarction or death [147]. Although their adaptation is not universal, contemporary evidence also suggests a mortality benefit, at the cost of increased bleeding risk [148]. Interestingly, a recent meta-analysis demonstrated an efficacious profile of the addition of GP inhibitors in the difficult-to-manage scenario of patients with an acute myocardial infarction complicated with cardiogenic shock [149]. Regarding ischemic strokes, GP inhibitors, were not met with efficacy while the risk of intracranial bleeding was augmented, driven by abciximab [150]. Tirofiban and eptifibatide in low doses may be considered safe in stroke treatment, as recently reported [151]. Although their use has dropped following the implementation of P2Y_12_ inhibitors in routine clinical practice, this drug category could still be important in cases of P2Y_12_ inhibitor intolerance or in patients at high risk for thrombotic complications.

#### 4.1.4. PAR-1 Antagonism

Vorapaxar is a novel agent introduced as a competitive, high-affinity PAR-1 inhibitor. It is metabolized by CYP3A4 and primarily excreted fecally. Initial preclinical data indicated a potent attenuation of agonist-induced platelet activation following its use [152]. In phase III trials Thrombin Receptor Antagonist Clinical Event Reduction in acute coronary syndrome (TRACER) and Thrombin Receptor Antagonist in Secondary Prevention of Atherothrombotic Ischaemic Events-TIMI 50 (TRA-2P-TIMI 50) a benefit was noted in specific patient populations such as those with prior myocardial infarction or peripheral arterial disease [153,154]. A net benefit may also be observed in patients with diabetes mellitus [155], chronic kidney disease [156], or prior coronary stenting [157]. It should be noted, however, that a high risk of intracranial bleeding was consistently reported [153,154].

#### 4.1.5. Caplacizumab

Caplacizumab, initially labeled as ALX-0081, is a humanized nanobody that potently binds to the A1 domain of VWF, inhibiting the GP1b/IX/V receptor [158]. Its major indication is the management of acquired thrombotic thrombocytopenic purpura, a condition which is characterized by decreased ADAMTS13 activity and thus defective breakdown of ultrahigh VWF multimers. When patients with acquired thrombotic thrombocytopenic purpura were treated with caplacizumab or placebo added to the standard of care in the TITAN and HERCULES trials, the study group experienced a faster normalization of platelet counts and resolution of the episode compared to the control group, an effect which was maintained throughout the treatment period [159,160]. Interestingly, significantly fewer thrombotic events compared to controls in the TITAN trial [161], a finding which was, however, not confirmed in the HERCULES trial [160]. This anti-thrombotic effect has been further tested, with in vitro evidence suggesting that this agent inhibited platelet activation and adhesion, with similar potency and higher safety compared to other antiplatelet agents [158]. Moreover, its antiplatelet effect may not be altered by concomitant antithrombotic use, as shown through the addition of caplacizumab of blood from patients undergoing elective revascularization who were receiving aspirin, clopidogrel, and heparin [162]. The required concentration was, however, related to the levels of VWF [162]. Caplacizumab was additionally efficacious in the management of experimental middle cerebral artery thrombosis when administered shortly after the occlusion, also inducing reperfusion, with a superior safety profile compared to tirofiban or recombinant tissue plasminogen activator [163]. Additional evidence in the context of atherosclerotic diseases is needed to consider a possible indication for this group of patients.

#### 4.1.6. Direct Oral Anticoagulants

Although anticoagulants primarily aim at the inhibition of the coagulation cascade, their secondary effect concerns the inhibition of platelet activation. Their efficacy in the setting of atherosclerotic diseases has been recently proven [164]. Dabigatran in particular may attenuate the process of platelet activation as shown in thrombin-stimulated platelets where dabigatran inhibited CD62 [165]. This platelet inhibitory effect appears to be dose-dependent [166]. Interestingly, cessation of dabigatran may induce a state of platelet activation [167]. Those antiplatelet effects have also been proposed for the factor Xa inhibitors [168]. It should be stressed that conflicting results have been reported, with Arantes et al. noting no differences in the platelet function of patients with coronary artery disease when treated with dabigatran [169]. An in-vitro study by Jourdi et al. found more potent antiplatelet effects with dabigatran than with rivaroxaban [170]. Thus, continuing research in the antiplatelet effects of direct oral anticoagulants is warranted.

### 4.2. Off-Target Antiplatelet Drugs

The potential effect of available anti-inflammatory medication on inhibition of platelet activation has been a topic of scientific interest. Beginning with colchicine, whose mechanism of action involves the inhibition of core inflammatory mediators such as IL-1 and NLRP3 inflammasome, its administration in healthy individuals led to reduction in platelet activation markers, such as P-selectin and platelet-neutrophil complexes, without any effect on platelet aggregation, however [171]. Future studies in the setting of coronary artery disease have demonstrated an additional anti-aggregatory action when platelet activation was induced by agonists [172,173]. A recent study by Pennings et al. revealed the modulation of GPVI receptor pathway as the antiplatelet mechanism of action of colchicine [174]. It could be those additional antiplatelet effects that mediate the protective effect of colchicine in atherosclerotic diseases as evidenced in large randomized clinical trials [175]. Moving to TNF-α inhibitors, there is contradictory evidence concerning their effect on platelet activation, with a study revealing a possible antiplatelet effect [176], while others failed to validate such actions [177,178]. At the same time, the IL-6 inhibitor tocilizumab may attenuate platelet activation and aggregation, since its use was accompanied by a reduction in P-selectin and platelet-neutrophil aggregates [178,179]. The IL-6 inhibitor canakinumab led to a significant reduction of atherosclerotic events in the large-scale randomized clinical trial CANTOS of patients with a history of myocardial infarction and increased inflammatory burden [180]. However, little is known regarding its effect on platelet function [181]. Furthermore, latest evidence from experimental studies has highlighted the surprising antiplatelet effects of BTK inhibitors ibrutinib and acalabrutinib [182,183,184]. Interestingly, they may specifically block thrombus formation at the site of atherosclerotic plaques through the inhibition of GP1b and GPVI signaling, while preserving the essential platelet hemostasis [47]. However, a recent report suggested that BTK inhibitors could also be considered as CLEC2 inhibitors [184].

### 4.3. Drugs under Investigation

Investigation of agents with a potent antiplatelet activity together a favorable safety profile remains elusive and has been a topic of intense scientific research (Table 2).

Beginning with VWF inhibitors, they may attenuate the process of platelet activation as evidenced in studies of aptamers. Beginning with ARC1779, an aptamer binding to the A1 domain of VWF, inhibition of VWF-induced platelet activation in vitro together with attenuation of thrombus formation in vivo was initially observed [185]. No effects on ADP-, collagen-, or AA-induced platelet activation were detected, indicating the selectivity of this agent towards VWF inhibition [186]. In a phase I trial, a dose- and concentration-dependent inhibition of platelet function was noted [187]. Its use was further tested in a randomized trial of 36 patients undergoing carotid endarterectomy, where embolic signal counts -evaluated by transcranial doppler- were correlated to VWF inhibition at the expense of increased perioperative bleeding [188]. A novel aptamer (TAGX-0004) may possess more potent antiplatelet activity than ARC1779 and comparable to caplacizumab, as recently described [189]. As far as BT200 is concerned, it is a 3rd generation aptamer against VWF which inhibited ristocetin-induced platelet aggregation after stimulation with desmopressin [190]. BT200 also lowered the VWF activity in plasma of patients with an acute coronary syndrome or stroke in a concentration-dependent manner [191,192]. In the recently reported phase I trial in 112 healthy volunteers, the use of BT200 together with desmopressin resulted in dose-dependent inhibition of rostocetin-induced platelet aggregation, as well as affecting the shear-dependent platelet function [193]. Subsequent studies should provide additional clinical evidence for the use of BT200 in hematologic as well as in cardiovascular diseases. Lastly, the recently reported DTRI-031 anti-VWF aptamer is an additional approach towards platelet inhibition under investigation, with a significant advantage being the reversal ability of an oligonucleotide [194].

Moving to novel GP inhibitors, anfibatide, a direct anti-GP1b derived from snake venom, was able to inhibit ristocetin- and VWF-induced platelet adhesion and aggregation at high shear conditions, together with thrombolytic effects. It was also efficient in vivo, inhibiting the thrombotic process in VWF-deficient mice without affecting bleeding parameters [195]. Subsequent studies confirmed its effectiveness in the amelioration of acute cerebral ischemia and reperfusion injury in mice, comparable to tirofiban’s, with a more favorable safety profile [196]. The effect of this agent may be based on the preservation of the blood-brain-barrier integrity driven by the upregulation of Epac1 [197]. Concerning clinical trials, anfibatide inhibited VWF-induced platelet aggregation in healthy volunteers, an effect that lasted for 8 h, without alteration of coagulation parameters or serious adverse events [198]. In the subsequent phase IIa study of 90 patients with non-ST segment elevation myocardial infarction, anfibatide dose-dependently inhibited ex-vivo platelet aggregation, significantly higher compared to placebo [199]. Comparable revascularization and short-term clinical outcomes to placebo were reported [199]. A humanized monoclonal antibody against GP1b by the name of h6B4-Fab has also been developed, with only preclinical evidence of long-lasting, high-affinity binding to VWF [200].

GPVI inhibitors may also emerge as therapeutic tools in atherosclerotic diseases since GPVI is an important mediator of atherothrombosis [201]. Revacept, a fusion of the extracellular domain of GPVI with the human immunoglobulin Fc domain (GPVI-Fc), attenuated platelet aggregation in a mouse model of carotid artery thrombosis, and was also detected at the arterial lesions [202]. By competing with platelet GPVI for its binding sites on collagen, revacept produced similar outcomes (functionality, infarct size, edema) compared to thrombolytic therapy in a mouse model of middle cerebral artery occlusion, while their combination was superior [203]. However, revacept was not associated with intracranial hemorrhage [203]. When added on top of established antiplatelet agents (aspirin, ticagrelor) or abciximab in an in-vitro atherosclerotic condition, revacept further reduced plaque-induced platelet aggregation and enhanced the platelet inhibitory activity of the other agents, with no increase in the closure time assessed by a platelet function analyzer [204]. The combination of aspirin, ticagrelor, and revacept achieved nearly complete antiplatelet activity [204]. A fusion of GPVI-Fc with CD39, known for ADP inhibiting actions, resulted in attenuated ADP-, collagen- and atherosclerotic plaque-induced platelet aggregation and diminished thrombus formation, without any evidence of increased bleeding [205]. These findings were successfully validated in mouse model of arterial thrombosis [205]. However, the results of the recently reported ISAR-PLASTER phase 2 trial in 334 patients undergoing elective percutaneous revascularization for coronary artery disease were neutral, demonstrating no differences between the two dosing regimens of revacept compared to placebo regarding the composite endpoint of death or high-sensitivity cardiac troponin increase to at least five times the upper limit of normal within 48 h from randomization, despite the significant reduction of collagen-induced platelet aggregation in the high-dose revacept arm. Bleeding events did not differ significantly between the groups [206]. Another trial of revacept in patients with symptomatic carotid artery disease is currently ongoing [207], with its results being eagerly awaited. Glenzocimab, previously known as ACT017, is another representative of this category, able to dose-dependently inhibit collagen-induced platelet activation ex vivo without affecting bleeding diathesis [208]. This effect was validated in the subsequent phase I trial, with the antiplatelet effects being accompanied by no adverse effects in bleeding [162]. Complete inhibition of platelet activation was noted after the 6-h infusion, with residual 60% platelet inhibition at 12 h post infusion [209]. Furthermore, glenzocimab led to thrombus resolution both on a collagen and atherosclerotic plaque surface, with increased effect under increased wall shear rate [210]. Those encouraging results ought to be tested further in future studies, also involving patients with acute atherosclerotic manifestations.

Inhibition of CLEC2 represents a novel concept in the antiplatelet drug development due to the importance of this molecule in platelet activation. Preliminary results have shown that cobalt hematoporphyrin, an inhibitor of podoplanin binding to CLEC2, inhibited CLEC2-mediated platelet activation and prolonged thrombotic occlusion of the injured femoral artery in mice [211]. Similar actions were reported following the use of the CLEC2-podoplanin binding inhibitor 2CP, without any effect on platelet activation due to other agonists [212]. A monoclonal antibody against CLEC2 by the name of AYP1 has been developed and may compete for CLEC2 binding with podoplanin and katachine and attenuates CLEC2-induced platelet activation [23].

Novel anti-inflammatory agents aiming at NETs, TLRs, and NLRP3 inflammasome have also been assessed experimentally regarding an offset antiplatelet effect. Beginning with NET inhibitors, both DNAse and chloramidine resulted in diminished agonist-induced platelet activation and thrombus formation [213,214,215]. An important influence of TLR4 inhibitors eritoran and resveratrol on platelet activation parameters were noted, since they were able to attenuate TRAP- and collagen-induced platelet aggregation, platelet-leukocyte aggregate formation, and the release of soluble CD40 ligand and platelet factor 4 [216,217]. A novel resveratrol analogue in the name of pterostilbene was also able to induce strong antiplatelet effects [218]. The selective NLRP3 inflammasome inhibitor MCC950 may attenuate platelet activation as observed in the studies of Vogel et al. and Cornelius et al. [46,219]. Recently, Wang et al. demonstrated the utility of MCC950 in bone marrow tissue after a myocardial infarction. After documenting the upregulation of NLPR3 inflammasome and the consequent IL-1β secretion, they administered MCC950 which resulted in impaired bone marrow megakaryocyte concentration and maturation, thus limiting platelet production. The survival benefit of the myocardial infarction mouse model treated with MCC950 could be stemming from a combination of diminished platelet activity with attenuated inflammatory responses [220]. Further research is required in the field of novel anti-inflammatory therapies since the data is mostly preliminary. Last but not least, therapeutic modulation of miRs might be an effective antiplatelet approach, bearing in mind the possible role of miRs in the regulation of platelet activation. In the study of Garcia et al., transfection of CD34^+^ megakaryocytes with miR-126-3p resulted in the formation of more reactive platelet-like structures compared to the control group due to an effect in actin-regulating gene plexin B2 [98]. Their study group also performed megakaryocyte transfection with miR-204-5p, which also led to the production of hyperreactive platelet-like structures through the regulation of fibrinogen receptor by downregulating CDC42 [221].

## 5. Conclusions and Future Directions

Platelet activation, although critical in the case of vascular injury, is a well-characterized feature of various pathologic states, primarily atherosclerotic cardiovascular diseases. Several factors have been implicated in this process, with inflammation, endothelial dysfunction, and miRs being among its regulators. Important progress has been made in the field of the pharmacologic inhibition of platelet activation, including aspirin, P2Y_12_ inhibitors, and glycoprotein IIb/IIIa inhibitors. Moreover, secondary antiplatelet effects of direct oral anticoagulants and offset antiplatelet actions of anti-inflammatory, either existing or experimental, have been investigated in the recent years. Novel VWF and GP inhibitors are in various phases of development and upcoming clinical trials should clarify their role in the management of thrombotic diseases involving platelet activation. Lastly, the role of miRs in platelet activation deserves further research as they could end up being critical biomarkers or even treatment targets in this regard.

## Figures and Tables

**Table 1:** Potential inflammatory, endothelial, and miR mediators of platelet activation.

Inflammation	Endothelial Dysfunction	MiRs
Interleukins	↓ NO bioavailability	miR-126-3p
TNF-α	Thrombomodulin	miR-223
NLRP3 inflammasome	CD39	miR-320c
BTK	CD73	miR-181
PLT-NEU interactions	PGI_2_-TXA_2_ imbalance	miR-26b
NETs	VWF	miR-15b-5p
TLRs	Endothelial glycocalyx	miR-93
		miR-150-5p
		miR-423-3p
		miR-1180-3p
		miR-204-5p

TNF: tumor necrosis factor, NLRP3: NLR family pyrin domain-containing 3, BTK: Bruton’s tyrosine kinase, PLT: platelet, NEU: neutrophil, NET: neutrophil extracellular traps, TLR: toll-like receptor, NO: nitric oxide, PGI2: prostacyclin, TXA2: thromboxane A2, vWF: von Willebrand Factor. ↓ denotes reduction.

**Table 2:** Summary of the effects of investigational antiplatelet agents.

Agent	Target	Preclinical/Clinical Evidence
ARC1779	VWF (A1 domain)	↓ VWF-induced platelet activation (in vitro) No effect on ADP-, collagen-, or AA-induced platelet activation (in vitro) ↓ platelet activation, dose- and concentration dependent (in humans) ↓ embolic signals proportional to VWF inhibition in carotid endarterectomy (in humans) ↑ Perioperative bleeding in carotid endarterectomy (in humans)
TAGX-004	VWF (A1 domain)	↑ antiplatelet activity compared to ARC1779 (in vitro)
BT200	VWF (A1 domain)	↓ ristocetin-induced platelet aggregation (ex vivo and in humans) ↓ VWF activity in patients with an ACS or stroke (in humans)
DTRI-031	VWF (A1 domain)	↓ collagen- and ADP-induced platelet activation (ex vivo) Prevention of carotid artery thrombosis (in vivo) Induction of recanalization in carotid artery occlusion (in vivo)
Anfibatide	GP1b	↓ ristocetin- and VWF-induced platelet adhesion and aggregation (in vitro and in humans) ↓ thrombosis in VWF-deficient mice (in vivo) ↓ activation of platelets from patients with an ACS, without any effect on revascularization parameters or myocardial injury markers (in humans) No increases in bleeding
h6B4-Fab	GP1b	↓ cyclic flow reductions in stenosed femoral artery (in vivo)
Revacept	GPVI	↓ platelet aggregation (in vivo) Comparable functional outcomes, infarct size, edema to thrombolytic therapy in cerebral ischemia (in vivo) ↑ platelet inhibition of other antiplatelets when used in combination (in vitro) No difference in death or high-sensitivity cardiac troponin increase in patients undergoing elective PCI for CAD (in humans) No increases in bleeding (in humans)
Glenzocimab	GPVI	↓ collagen-induced platelet activation (ex vivo) Thrombus resolution, enhanced by increased wall shear rate (in vitro) Complete inhibition of platelet activation was noted after infusion, 60% platelet inhibition maintained 12 h post infusion (in humans) No increases in bleeding (in humans)
Cobalt hematoporphyrin	CLEC2	↓ CLEC2-induced platelet activation (in vitro) Prolonged time to occlusion of injured femoral artery (in vivo)
2CP	CLEC2	↓ CLEC2-induced platelet activation (in vitro)
AYP1	CLEC2	↓ CLEC2-induced platelet activation (in vitro)

VWF: von Willebrand factor, ADP: adenosine diphosphate, AA: arachidonic acid, ACS: acute coronary syndrome, GP: glycoprotein, PCI: percutaneous coronary intervention, CAD: coronary artery disease. ↓ denotes reduction/inhibition, ↑ denotes increase/stimulation.

## Data Availability

Not applicable.

## References

[1] Kuter D.J. (2013). The biology of thrombopoietin and thrombopoietin receptor agonists. Int. J. Hematol..

[2] Behrens K., Alexander W.S. (2018). Cytokine control of megakaryopoiesis. Growth Factors.

[3] Hitchcock I.S., Kaushansky K. (2014). Thrombopoietin from beginning to end. Br. J. Haematol..

[4] Kaser A., Brandacher G., Steurer W., Kaser S., Offner F.A., Zoller H., Theurl I., Widder W., Molnar C., Ludwiczek O. (2001). Interleukin-6 stimulates thrombopoiesis through thrombopoietin: Role in inflammatory thrombocytosis. Blood.

[5] Kanaji T., Vo M.N., Kanaji S., Zarpellon A., Shapiro R., Morodomi Y., Yuzuriha A., Eto K., Belani R., Do M.H. (2018). Tyrosyl-tRNA synthetase stimulates thrombopoietin-independent hematopoiesis accelerating recovery from thrombocytopenia. Proc. Natl. Acad. Sci. USA.

[6] Noetzli L.J., French S.L., Machlus K.R. (2019). New Insights Into the Differentiation of Megakaryocytes From Hematopoietic Progenitors. Arterioscler. Thromb. Vasc. Biol..

[7] Boscher J., Guinard I., Eckly A., Lanza F., Leon C. (2020). Blood platelet formation at a glance. J. Cell Sci..

[8] Schachtner H., Calaminus S.D., Sinclair A., Monypenny J., Blundell M.P., Leon C., Holyoake T.L., Thrasher A.J., Michie A.M., Vukovic M. (2013). Megakaryocytes assemble podosomes that degrade matrix and protrude through basement membrane. Blood.

[9] Eckly A., Scandola C., Oprescu A., Michel D., Rinckel J.Y., Proamer F., Hoffmann D., Receveur N., Leon C., Bear J.E. (2020). Megakaryocytes use in vivo podosome-like structures working collectively to penetrate the endothelial barrier of bone marrow sinusoids. J. Thromb. Haemost..

[10] Brown E., Carlin L.M., Nerlov C., Lo Celso C., Poole A.W. (2018). Multiple membrane extrusion sites drive megakaryocyte migration into bone marrow blood vessels. Life. Sci. Alliance.

[11] Lefrancais E., Looney M.R. (2019). Platelet Biogenesis in the Lung Circulation. Physiology.

[12] Ouzegdouh Y., Capron C., Bauer T., Puymirat E., Diehl J.L., Martin J.F., Cramer-Borde E. (2018). The physical and cellular conditions of the human pulmonary circulation enable thrombopoiesis. Exp. Hematol..

[13] Holinstat M. (2017). Normal platelet function. Cancer Metastasis Rev..

[14] Tomaiuolo M., Brass L.F., Stalker T.J. (2017). Regulation of Platelet Activation and Coagulation and Its Role in Vascular Injury and Arterial Thrombosis. Interv. Cardiol. Clin..

[15] Huang J., Li X., Shi X., Zhu M., Wang J., Huang S., Huang X., Wang H., Li L., Deng H. (2019). Platelet integrin alphaIIbbeta3: Signal transduction, regulation, and its therapeutic targeting. J. Hematol. Oncol..

[16] Hottz E.D., Bozza F.A., Bozza P.T. (2018). Platelets in Immune Response to Virus and Immunopathology of Viral Infections. Front. Med..

[17] McDonald B., Dunbar M. (2019). Platelets and Intravascular Immunity: Guardians of the Vascular Space During Bloodstream Infections and Sepsis. Front. Immunol..

[18] Claushuis T.A.M., Van Der Veen A.I.P., Horn J., Schultz M.J., Houtkooper R.H., Van’t Veer C., Van Der Poll T. (2019). Platelet Toll-like receptor expression and activation induced by lipopolysaccharide and sepsis. Platelets.

[19] Badimon L., Suades R., Fuentes E., Palomo I., Padro T. (2016). Role of Platelet-Derived Microvesicles As Crosstalk Mediators in Atherothrombosis and Future Pharmacology Targets: A Link between Inflammation, Atherosclerosis, and Thrombosis. Front. Pharm..

[20] Louwette S., Van Geet C., Freson K. (2012). Regulators of G protein signaling: Role in hematopoiesis, megakaryopoiesis and platelet function. J. Thromb. Haemost..

[21] Senis Y.A., Mazharian A., Mori J. (2014). Src family kinases: At the forefront of platelet activation. Blood.

[22] Makhoul S., Kumm E., Zhang P., Walter U., Jurk K. (2020). The Serine/Threonine Protein Phosphatase 2A (PP2A) Regulates Syk Activity in Human Platelets. Int. J. Mol. Sci..

[23] Moran L.A., Di Y., Sowa M.A., Hermida-Nogueira L., Barrachina M.N., Martin E., Mize T.H., Clark J.C., Eble J.A., Moreira D. (2022). Katacine is a new ligand of CLEC-2 that acts as a platelet agonist. Thromb. Haemost..

[24] Badolia R., Inamdar V., Manne B.K., Dangelmaier C., Eble J.A., Kunapuli S.P. (2017). Gq pathway regulates proximal C-type lectin-like receptor-2 (CLEC-2) signaling in platelets. J. Biol. Chem..

[25] Navarro-Nunez L., Langan S.A., Nash G.B., Watson S.P. (2013). The physiological and pathophysiological roles of platelet CLEC-2. Thromb. Haemost..

[26] Koupenova M., Ravid K. (2018). Biology of Platelet Purinergic Receptors and Implications for Platelet Heterogeneity. Front. Pharm..

[27] Van Kolen K., Slegers H. (2006). Integration of P2Y receptor-activated signal transduction pathways in G protein-dependent signalling networks. Purinergic Signal..

[28] Jin J., Mao Y., Thomas D., Kim S., Daniel J.L., Kunapuli S.P. (2009). RhoA downstream of G(q) and G(12/13) pathways regulates protease-activated receptor-mediated dense granule release in platelets. Biochem. Pharm..

[29] Kahn M.L., Nakanishi-Matsui M., Shapiro M.J., Ishihara H., Coughlin S.R. (1999). Protease-activated receptors 1 and 4 mediate activation of human platelets by thrombin. J. Clin. Investig..

[30] Flaumenhaft R. (2003). Molecular basis of platelet granule secretion. Arter. Thromb. Vasc. Biol..

[31] De Jong J.S., Dekker L.R. (2010). Platelets and cardiac arrhythmia. Front. Physiol..

[32] Fernandez D.I., Kuijpers M.J.E., Heemskerk J.W.M. (2021). Platelet calcium signaling by G-protein coupled and ITAM-linked receptors regulating anoctamin-6 and procoagulant activity. Platelets.

[33] Sagris M., Theofilis P., Antonopoulos A.S., Oikonomou E., Paschaliori C., Galiatsatos N., Tsioufis K., Tousoulis D. (2021). Inflammation in Coronary Microvascular Dysfunction. Int. J. Mol. Sci..

[34] Sagris M., Theofilis P., Antonopoulos A.S., Tsioufis C., Oikonomou E., Antoniades C., Crea F., Kaski J.C., Tousoulis D. (2021). Inflammatory Mechanisms in COVID-19 and Atherosclerosis: Current Pharmaceutical Perspectives. Int. J. Mol. Sci..

[35] Theofilis P., Sagris M., Oikonomou E., Antonopoulos A.S., Siasos G., Tsioufis C., Tousoulis D. (2021). Inflammatory Mechanisms Contributing to Endothelial Dysfunction. Biomedicines.

[36] Theofilis P., Sagris M., Antonopoulos A.S., Oikonomou E., Tsioufis C., Tousoulis D. (2021). Inflammatory Mediators of Platelet Activation: Focus on Atherosclerosis and COVID-19. Int. J. Mol. Sci..

[37] Oikonomou E., Leopoulou M., Theofilis P., Antonopoulos A.S., Siasos G., Latsios G., Mystakidi V.C., Antoniades C., Tousoulis D. (2020). A link between inflammation and thrombosis in atherosclerotic cardiovascular diseases: Clinical and therapeutic implications. Atherosclerosis.

[38] Beaulieu L.M., Lin E., Mick E., Koupenova M., Weinberg E.O., Kramer C.D., Genco C.A., Tanriverdi K., Larson M.G., Benjamin E.J. (2014). Interleukin 1 receptor 1 and interleukin 1beta regulate megakaryocyte maturation, platelet activation, and transcript profile during inflammation in mice and humans. Arter. Thromb. Vasc. Biol..

[39] Marta R.F., Goette N.P., Lev P.R., Chazarreta C.D., Pirola C.J., Molinas F.C. (2005). Normal platelets possess the soluble form of IL-6 receptor. Cytokine.

[40] Regnault V., De Maistre E., Carteaux J.P., Gruel Y., Nguyen P., Tardy B., Lecompte T. (2003). Platelet activation induced by human antibodies to interleukin-8. Blood.

[41] Page M.J., Bester J., Pretorius E. (2018). Interleukin-12 and its procoagulant effect on erythrocytes, platelets and fibrin(ogen): The lesser known side of inflammation. Br. J. Haematol..

[42] Bester J., Pretorius E. (2016). Effects of IL-1beta, IL-6 and IL-8 on erythrocytes, platelets and clot viscoelasticity. Sci. Rep..

[43] Davizon-Castillo P., McMahon B., Aguila S., Bark D., Ashworth K., Allawzi A., Campbell R.A., Montenont E., Nemkov T., D’Alessandro A. (2019). TNF-alpha-driven inflammation and mitochondrial dysfunction define the platelet hyperreactivity of aging. Blood.

[44] Hottz E.D., Monteiro A.P., Bozza F.A., Bozza P.T. (2015). Inflammasome in platelets: Allying coagulation and inflammation in infectious and sterile diseases?. Mediat. Inflamm..

[45] Brown G.T., Narayanan P., Li W., Silverstein R.L., McIntyre T.M. (2013). Lipopolysaccharide stimulates platelets through an IL-1beta autocrine loop. J. Immunol..

[46] Vogel S., Kamimura S., Arora T., Smith M.L., Almeida L.E.F., Combs C.A., Thein S.L., Quezado Z.M.N. (2021). NLRP3 inflammasome and bruton tyrosine kinase inhibition interferes with upregulated platelet aggregation and in vitro thrombus formation in sickle cell mice. Biochem. Biophys. Res. Commun..

[47] Busygina K., Jamasbi J., Seiler T., Deckmyn H., Weber C., Brandl R., Lorenz R., Siess W. (2018). Oral Bruton tyrosine kinase inhibitors selectively block atherosclerotic plaque-triggered thrombus formation in humans. Blood.

[48] Zucoloto A.Z., Jenne C.N. (2019). Platelet-Neutrophil Interplay: Insights Into Neutrophil Extracellular Trap (NET)-Driven Coagulation in Infection. Front. Cardiovasc. Med..

[49] Rigg R.A., Healy L.D., Chu T.T., Ngo A.T.P., Mitrugno A., Zilberman-Rudenko J., Aslan J.E., Hinds M.T., Vecchiarelli L.D., Morgan T.K. (2019). Protease-activated receptor 4 activity promotes platelet granule release and platelet-leukocyte interactions. Platelets.

[50] Seif K., Alidzanovic L., Tischler B., Ibrahim N., Zagrapan B., Rauscher S., Salzmann M., Hell L., Mauracher L.M., Budde U. (2018). Neutrophil-Mediated Proteolysis of Thrombospondin-1 Promotes Platelet Adhesion and String Formation. Thromb. Haemost..

[51] Quinn K.L., Henriques M., Tabuchi A., Han B., Yang H., Cheng W.E., Tole S., Yu H., Luo A., Charbonney E. (2011). Human neutrophil peptides mediate endothelial-monocyte interaction, foam cell formation, and platelet activation. Arter. Thromb. Vasc. Biol..

[52] Horn M., Bertling A., Brodde M.F., Muller A., Roth J., Van Aken H., Jurk K., Heilmann C., Peters G., Kehrel B.E. (2012). Human neutrophil alpha-defensins induce formation of fibrinogen and thrombospondin-1 amyloid-like structures and activate platelets via glycoprotein IIb/IIIa. J. Thromb. Haemost..

[53] Kaiser P., Harenberg J., Walenga J.M., Huhle G., Giese C., Prechel M., Hoppensteadt D., Fareed J. (2001). Effects of a heparin-binding protein on blood coagulation and platelet function. Semin. Thromb. Hemost..

[54] Santilli F., Paloscia L., Liani R., Di Nicola M., Di Marco M., Lattanzio S., La Barba S., Pascale S., Mascellanti M., Davi G. (2014). Circulating myeloid-related protein-8/14 is related to thromboxane-dependent platelet activation in patients with acute coronary syndrome, with and without ongoing low-dose aspirin treatment. J. Am. Heart Assoc..

[55] Liang X., Xiu C., Liu M., Lin C., Chen H., Bao R., Yang S., Yu J. (2019). Platelet-neutrophil interaction aggravates vascular in fl ammation and promotes the progression of atherosclerosis by activating the TLR4/NF-kappaB pathway. J. Cell Biochem..

[56] Pircher J., Czermak T., Ehrlich A., Eberle C., Gaitzsch E., Margraf A., Grommes J., Saha P., Titova A., Ishikawa-Ankerhold H. (2018). Cathelicidins prime platelets to mediate arterial thrombosis and tissue inflammation. Nat. Commun..

[57] Faraday N., Schunke K., Saleem S., Fu J., Wang B., Zhang J., Morrell C., Dore S. (2013). Cathepsin G-dependent modulation of platelet thrombus formation in vivo by blood neutrophils. PLoS ONE.

[58] Rossaint J., Kuhne K., Skupski J., Van Aken H., Looney M.R., Hidalgo A., Zarbock A. (2016). Directed transport of neutrophil-derived extracellular vesicles enables platelet-mediated innate immune response. Nat. Commun..

[59] Thakur M., Evans B., Schindewolf M., Baumgartner I., Doring Y. (2021). Neutrophil Extracellular Traps Affecting Cardiovascular Health in Infectious and Inflammatory Diseases. Cells.

[60] Noubouossie D.F., Whelihan M.F., Yu Y.B., Sparkenbaugh E., Pawlinski R., Monroe D.M., Key N.S. (2017). In vitro activation of coagulation by human neutrophil DNA and histone proteins but not neutrophil extracellular traps. Blood.

[61] Semeraro F., Ammollo C.T., Morrissey J.H., Dale G.L., Friese P., Esmon N.L., Esmon C.T. (2011). Extracellular histones promote thrombin generation through platelet-dependent mechanisms: Involvement of platelet TLR2 and TLR4. Blood.

[62] De Boer O.J., Li X., Teeling P., Mackaay C., Ploegmakers H.J., van der Loos C.M., Daemen M.J., de Winter R.J., van der Wal A.C. (2013). Neutrophils, neutrophil extracellular traps and interleukin-17 associate with the organisation of thrombi in acute myocardial infarction. Thromb. Haemost..

[63] Laridan E., Denorme F., Desender L., Francois O., Andersson T., Deckmyn H., Vanhoorelbeke K., De Meyer S.F. (2017). Neutrophil extracellular traps in ischemic stroke thrombi. Ann. Neurol..

[64] Rivadeneyra L., Carestia A., Etulain J., Pozner R.G., Fondevila C., Negrotto S., Schattner M. (2014). Regulation of platelet responses triggered by Toll-like receptor 2 and 4 ligands is another non-genomic role of nuclear factor-kappaB. Thromb. Res..

[65] Kalvegren H., Skoglund C., Helldahl C., Lerm M., Grenegard M., Bengtsson T. (2010). Toll-like receptor 2 stimulation of platelets is mediated by purinergic P2X1-dependent Ca2+ mobilisation, cyclooxygenase and purinergic P2Y1 and P2Y12 receptor activation. Thromb. Haemost..

[66] Klarstrom Engstrom K., Brommesson C., Kalvegren H., Bengtsson T. (2014). Toll like receptor 2/1 mediated platelet adhesion and activation on bacterial mimetic surfaces is dependent on src/Syk-signaling and purinergic receptor P2X1 and P2Y12 activation. Biointerphases.

[67] Lopes Pires M.E., Clarke S.R., Marcondes S., Gibbins J.M. (2017). Lipopolysaccharide potentiates platelet responses via toll-like receptor 4-stimulated Akt-Erk-PLA2 signalling. PLoS ONE.

[68] Stahl A.L., Svensson M., Morgelin M., Svanborg C., Tarr P.I., Mooney J.C., Watkins S.L., Johnson R., Karpman D. (2006). Lipopolysaccharide from enterohemorrhagic Escherichia coli binds to platelets through TLR4 and CD62 and is detected on circulating platelets in patients with hemolytic uremic syndrome. Blood.

[69] Hally K.E., La Flamme A.C., Larsen P.D., Harding S.A. (2017). Platelet Toll-like receptor (TLR) expression and TLR-mediated platelet activation in acute myocardial infarction. Thromb. Res..

[70] De Stoppelaar S.F., Claushuis T.A., Schaap M.C., Hou B., van der Poll T., Nieuwland R., van’t Veer C. (2016). Toll-Like Receptor Signalling Is Not Involved in Platelet Response to Streptococcus pneumoniae In Vitro or In Vivo. PLoS ONE.

[71] D’Atri L.P., Etulain J., Rivadeneyra L., Lapponi M.J., Centurion M., Cheng K., Yin H., Schattner M. (2015). Expression and functionality of Toll-like receptor 3 in the megakaryocytic lineage. J. Thromb. Haemost..

[72] Rex S., Beaulieu L.M., Perlman D.H., Vitseva O., Blair P.S., McComb M.E., Costello C.E., Freedman J.E. (2009). Immune versus thrombotic stimulation of platelets differentially regulates signalling pathways, intracellular protein-protein interactions, and alpha-granule release. Thromb. Haemost..

[73] Smolenski A. (2012). Novel roles of cAMP/cGMP-dependent signaling in platelets. J. Thromb. Haemost..

[74] Szabo C., Ischiropoulos H., Radi R. (2007). Peroxynitrite: Biochemistry, pathophysiology and development of therapeutics. Nat. Rev. Drug Discov..

[75] Schwarz U.R., Walter U., Eigenthaler M. (2001). Taming platelets with cyclic nucleotides. Biochem. Pharm..

[76] Cheng Y., Austin S.C., Rocca B., Koller B.H., Coffman T.M., Grosser T., Lawson J.A., FitzGerald G.A. (2002). Role of prostacyclin in the cardiovascular response to thromboxane A2. Science.

[77] Koupenova M., Kehrel B.E., Corkrey H.A., Freedman J.E. (2017). Thrombosis and platelets: An update. Eur. Heart J..

[78] Watanabe-Kusunoki K., Nakazawa D., Ishizu A., Atsumi T. (2020). Thrombomodulin as a Physiological Modulator of Intravascular Injury. Front. Immunol..

[79] Nightingale T., Cutler D. (2013). The secretion of von Willebrand factor from endothelial cells; an increasingly complicated story. J. Thromb. Haemost..

[80] Blair P., Flaumenhaft R. (2009). Platelet alpha-granules: Basic biology and clinical correlates. Blood Rev..

[81] Wu M.D., Atkinson T.M., Lindner J.R. (2017). Platelets and von Willebrand factor in atherogenesis. Blood.

[82] Dong J.F., Moake J.L., Nolasco L., Bernardo A., Arceneaux W., Shrimpton C.N., Schade A.J., McIntire L.V., Fujikawa K., Lopez J.A. (2002). ADAMTS-13 rapidly cleaves newly secreted ultralarge von Willebrand factor multimers on the endothelial surface under flowing conditions. Blood.

[83] Alphonsus C.S., Rodseth R.N. (2014). The endothelial glycocalyx: A review of the vascular barrier. Anaesthesia.

[84] Safiah Mokhtar S., Vanhoutte P.M., Leung S.W.S., Imran Yusof M., Wan Sulaiman W.A., Zaharil Mat Saad A., Suppian R., Ghulam Rasool A.H. (2013). Reduced expression of prostacyclin synthase and nitric oxide synthase in subcutaneous arteries of type 2 diabetic patients. Tohoku J. Exp. Med..

[85] Roy C., Tabiasco J., Caillon A., Delneste Y., Merot J., Favre J., Guihot A.L., Martin L., Nascimento D.C., Ryffel B. (2018). Loss of vascular expression of nucleoside triphosphate diphosphohydrolase-1/CD39 in hypertension. Purinergic Signal..

[86] Takahashi-Sato K., Murakawa M., Kimura J., Ito M.A., Matsuoka I. (2013). Loss of ectonucleotidases from the coronary vascular bed after ischemia-reperfusion in isolated rat heart. BMC Cardiovasc. Disord..

[87] Uchimido R., Schmidt E.P., Shapiro N.I. (2019). The glycocalyx: A novel diagnostic and therapeutic target in sepsis. Crit. Care.

[88] Becker B.F., Jacob M., Leipert S., Salmon A.H., Chappell D. (2015). Degradation of the endothelial glycocalyx in clinical settings: Searching for the sheddases. Br. J. Clin. Pharm..

[89] Lukasz A., Hillgruber C., Oberleithner H., Kusche-Vihrog K., Pavenstadt H., Rovas A., Hesse B., Goerge T., Kumpers P. (2017). Endothelial glycocalyx breakdown is mediated by angiopoietin-2. Cardiovasc. Res..

[90] Constantinescu A.A., Vink H., Spaan J.A. (2003). Endothelial cell glycocalyx modulates immobilization of leukocytes at the endothelial surface. Arter. Thromb. Vasc. Biol..

[91] Becker B.F., Chappell D., Bruegger D., Annecke T., Jacob M. (2010). Therapeutic strategies targeting the endothelial glycocalyx: Acute deficits, but great potential. Cardiovasc. Res..

[92] Reitsma S., Oude Egbrink M.G., Heijnen V.V., Megens R.T., Engels W., Vink H., Slaaf D.W., Van Zandvoort M.A. (2011). Endothelial glycocalyx thickness and platelet-vessel wall interactions during atherogenesis. Thromb. Haemost..

[93] Sempere L.F., Azmi A.S., Moore A. (2021). microRNA-based diagnostic and therapeutic applications in cancer medicine. Wiley Interdiscip. Rev. RNA.

[94] Theofilis P., Oikonomou E., Vogiatzi G., Antonopoulos A.S., Siasos G., Iliopoulos D.C., Perrea D., Tsioufis C., Tousoulis D. (2021). The impact of proangiogenic microRNA modulation on blood flow recovery following hind limb ischemia. A systematic review and meta-analysis of animal studies. Vasc. Pharm..

[95] Theofilis P., Vogiatzi G., Oikonomou E., Gazouli M., Siasos G., Katifelis H., Perrea D., Vavuranakis M., Iliopoulos D.C., Tsioufis C. (2021). The Effect of MicroRNA-126 Mimic Administration on Vascular Perfusion Recovery in an Animal Model of Hind Limb Ischemia. Front. Mol. Biosci..

[96] Zhou S.S., Jin J.P., Wang J.Q., Zhang Z.G., Freedman J.H., Zheng Y., Cai L. (2018). miRNAS in cardiovascular diseases: Potential biomarkers, therapeutic targets and challenges. Acta Pharm. Sin..

[97] Choi J.L., Li S., Han J.Y. (2014). Platelet function tests: A review of progresses in clinical application. Biomed. Res. Int..

[98] Garcia A., Dunoyer-Geindre S., Zapilko V., Nolli S., Reny J.L., Fontana P. (2019). Functional Validation of microRNA-126-3p as a Platelet Reactivity Regulator Using Human Haematopoietic Stem Cells. Thromb. Haemost..

[99] Pordzik J., Pisarz K., De Rosa S., Jones A.D., Eyileten C., Indolfi C., Malek L., Postula M. (2018). The Potential Role of Platelet-Related microRNAs in the Development of Cardiovascular Events in High-Risk Populations, Including Diabetic Patients: A Review. Front. Endocrinol..

[100] Li S., Guo L.Z., Kim M.H., Han J.Y., Serebruany V. (2017). Platelet microRNA for predicting acute myocardial infarction. J. Thromb. Thrombolysis lm..

[101] Landry P., Plante I., Ouellet D.L., Perron M.P., Rousseau G., Provost P. (2009). Existence of a microRNA pathway in anucleate platelets. Nat. Struct. Mol. Biol..

[102] Delgado Lagos F., Elgheznawy A., Kyselova A., Meyer Zu Heringdorf D., Ratiu C., Randriamboavonjy V., Mann A.W., Fisslthaler B., Siragusa M., Fleming I. (2021). Secreted modular calcium-binding protein 1 binds and activates thrombin to account for platelet hyperreactivity in diabetes. Blood.

[103] Leierseder S., Petzold T., Zhang L., Loyer X., Massberg S., Engelhardt S. (2013). MiR-223 is dispensable for platelet production and function in mice. Thromb. Haemost..

[104] Kaudewitz D., Skroblin P., Bender L.H., Barwari T., Willeit P., Pechlaner R., Sunderland N.P., Willeit K., Morton A.C., Armstrong P.C. (2016). Association of MicroRNAs and YRNAs With Platelet Function. Circ. Res..

[105] Dahiya N., Atreya C.D. (2019). RAP1 Downregulation by miR-320c Reduces Platelet Activation in Ex-vivo Storage. Microrna.

[106] Dahiya N., Atreya C.D. (2020). MiR-181a Reduces Platelet Activation via the Inhibition of Endogenous RAP1B. Microrna.

[107] Szilagyi B., Fejes Z., Poliska S., Pocsi M., Czimmerer Z., Patsalos A., Fenyvesi F., Rusznyak A., Nagy G., Kerekes G. (2020). Reduced miR-26b Expression in Megakaryocytes and Platelets Contributes to Elevated Level of Platelet Activation Status in Sepsis. Int. J. Mol. Sci..

[108] Becker K.C., Kwee L.C., Neely M.L., Grass E., Jakubowski J.A., Fox K.A.A., White H.D., Gregory S.G., Gurbel P.A., Carvalho L.P. (2020). Circulating MicroRNA Profiling in Non-ST Elevated Coronary Artery Syndrome Highlights Genomic Associations with Serial Platelet Reactivity Measurements. Sci. Rep..

[109] Pedersen O.B., Hvas A.M., Grove E.L., Larsen S.B., Pasalic L., Kristensen S.D., Nissen P.H. (2022). Association of whole blood microRNA expression with platelet function and turnover in patients with coronary artery disease. Thromb. Res..

[110] Garcia A., Dunoyer-Geindre S., Nolli S., Reny J.L., Fontana P. (2021). An Ex Vivo and In Silico Study Providing Insights into the Interplay of Circulating miRNAs Level, Platelet Reactivity and Thrombin Generation: Looking beyond Traditional Pharmacogenetics. J. Pers. Med..

[111] Tran J.Q.D., Pedersen O.H., Larsen M.L., Grove E.L., Kristensen S.D., Hvas A.M., Nissen P.H. (2020). Platelet microRNA expression and association with platelet maturity and function in patients with essential thrombocythemia. Platelets.

[112] Krammer T.L., Mayr M., Hackl M. (2020). microRNAs as promising biomarkers of platelet activity in antiplatelet therapy monitoring. Int. J. Mol. Sci..

[113] Willeit P., Zampetaki A., Dudek K., Kaudewitz D., King A., Kirkby N.S., Crosby-Nwaobi R., Prokopi M., Drozdov I., Langley S.R. (2013). Circulating microRNAs as novel biomarkers for platelet activation. Circ. Res..

[114] Braza-Boils A., Barwari T., Gutmann C., Thomas M.R., Judge H.M., Joshi A., Pechlaner R., Shankar-Hari M., Ajjan R.A., Sabroe I. (2020). Circulating MicroRNA Levels Indicate Platelet and Leukocyte Activation in Endotoxemia Despite Platelet P2Y12 Inhibition. Int. J. Mol. Sci..

[115] Carino A., De Rosa S., Sorrentino S., Polimeni A., Sabatino J., Caiazzo G., Torella D., Spaccarotella C., Mongiardo A., Strangio A. (2016). Modulation of Circulating MicroRNAs Levels during the Switch from Clopidogrel to Ticagrelor. Biomed. Res. Int..

[116] Chyrchel B., Toton-Zuranska J., Kruszelnicka O., Chyrchel M., Mielecki W., Kolton-Wroz M., Wolkow P., Surdacki A. (2015). Association of plasma miR-223 and platelet reactivity in patients with coronary artery disease on dual antiplatelet therapy: A preliminary report. Platelets.

[117] Shi R., Ge L., Zhou X., Ji W.J., Lu R.Y., Zhang Y.Y., Zeng S., Liu X., Zhao J.H., Zhang W.C. (2013). Decreased platelet miR-223 expression is associated with high on-clopidogrel platelet reactivity. Thromb. Res..

[118] Zhang Y.Y., Zhou X., Ji W.J., Shi R., Lu R.Y., Li J.L., Yang G.H., Luo T., Zhang J.Q., Zhao J.H. (2014). Decreased circulating microRNA-223 level predicts high on-treatment platelet reactivity in patients with troponin-negative non-ST elevation acute coronary syndrome. J. Thromb. Thrombolysis.

[119] Peng L., Liu J., Qin L., Liu J., Xi S., Lu C., Yin T. (2017). Interaction between platelet-derived microRNAs and CYP2C19*2 genotype on clopidogrel antiplatelet responsiveness in patients with ACS. Thromb. Res..

[120] Liu J., Qin L., Wang Z., Peng L., Liu J., Wang X., Du R., Zou Y., Wu Y., Yin T. (2020). Platelet-derived miRNAs as determinants of the antiplatelet response in clopidogrel-treated patients with ACS. Thromb. Res..

[121] Pedersen O.B., Grove E.L., Kristensen S.D., Nissen P.H., Hvas A.M. (2022). MicroRNA as Biomarkers for Platelet Function and Maturity in Patients with Cardiovascular Disease. Thromb. Haemost..

[122] Zheng S.L., Roddick A.J. (2019). Association of Aspirin Use for Primary Prevention With Cardiovascular Events and Bleeding Events: A Systematic Review and Meta-analysis. JAMA.

[123] Ma H., Gu Q., Niu H., Li X., Wang R. (2021). Benefits and Risks Associated With Aspirin Use in Patients With Diabetes for the Primary Prevention of Cardiovascular Events and Mortality: A Meta-Analysis. Front. Endocrinol..

[124] Masson W., Barbagelata L., Lavalle-Cobo A., Lobo M., Masson G., Nogueira J.P., Verges B. (2022). Low-doses aspirin in the primary prevention of cardiovascular disease in patients with diabetes: Meta-analysis stratified by baseline cardiovascular risk. Diabetes Metab. Syndr..

[125] Bates E.R., Lau W.C., Angiolillo D.J. (2011). Clopidogrel-drug interactions. J. Am. Coll. Cardiol..

[126] Condello F., Liccardo G., Ferrante G. (2021). Clinical Effects of Dual Antiplatelet Therapy or Aspirin Monotherapy after Acute Minor Ischemic Stroke or Transient Ischemic Attack, a Meta-Analysis. Curr. Pharm. Des..

[127] Squizzato A., Bellesini M., Takeda A., Middeldorp S., Donadini M.P. (2017). Clopidogrel plus aspirin versus aspirin alone for preventing cardiovascular events. Cochrane Database Syst. Rev..

[128] Liang L.R., Ma Q., Feng L., Qiu Q., Zheng W., Xie W.X. (2020). Long-term effect of clopidogrel in patients with and without diabetes: A systematic review and meta-analysis of randomized controlled trials. World J. Diabetes.

[129] Biswas M., Kali S.K. (2021). Association of CYP2C19 Loss-of-Function Alleles with Major Adverse Cardiovascular Events of Clopidogrel in Stable Coronary Artery Disease Patients Undergoing Percutaneous Coronary Intervention: Meta-analysis. Cardiovasc. Drugs Ther..

[130] Jafrin S., Naznin N.E., Reza M.S., Aziz M.A., Islam M.S. (2021). Risk of stroke in CYP2C19 LoF polymorphism carrier coronary artery disease patients undergoing clopidogrel therapy: An ethnicity-based updated meta-analysis. Eur. J. Intern. Med..

[131] Biswas M., Sukasem C., Khatun Kali M.S., Ibrahim B. (2022). Effects of the CYP2C19 LoF allele on major adverse cardiovascular events associated with clopidogrel in acute coronary syndrome patients undergoing percutaneous coronary intervention: A meta-analysis. Pharmacogenomics.

[132] Li J.L., Fu Y., Qin S.B., Liang G.K., Liu J., Nie X.Y., Chen J., Shi L.W., Shao H., Lu Y. (2018). Association between P2RY12 gene polymorphisms and adverse clinical events in coronary artery disease patients treated with clopidogrel: A systematic review and meta-analysis. Gene.

[133] Demcsak A., Lantos T., Balint E.R., Hartmann P., Vincze A., Bajor J., Czopf L., Alizadeh H., Gyongyi Z., Marta K. (2018). PPIs Are Not Responsible for Elevating Cardiovascular Risk in Patients on Clopidogrel-A Systematic Review and Meta-Analysis. Front. Physiol..

[134] Gupta R., Malik A.H., Briasoulis A., Joshi A.M., Guthier D.G., Popli T., Aronow W.S., Vyas A.V., Patel N.C., Ahmad H. (2021). Comparative Safety and Effectiveness of Loading Doses of P2Y12 Inhibitors in Patients Undergoing Elective PCI: A Network Meta-analysis. Cardiovasc. Drugs Ther..

[135] Navarese E.P., Khan S.U., Kolodziejczak M., Kubica J., Buccheri S., Cannon C.P., Gurbel P.A., De Servi S., Budaj A., Bartorelli A. (2020). Comparative Efficacy and Safety of Oral P2Y12 Inhibitors in Acute Coronary Syndrome: Network Meta-Analysis of 52 816 Patients From 12 Randomized Trials. Circulation.

[136] Farmakis I.T., Doundoulakis I., Zafeiropoulos S., Pagiantza A., Apostolidou-Kiouti F., Kourti O., Kassimis G., Haidich A.B., Karvounis H., Giannakoulas G. (2022). Comparative efficacy and safety of oral P2Y12 inhibitors for patients with chronic kidney disease and acute coronary syndrome: A network meta-analysis. Hell. J. Cardiol..

[137] Abusnina W., Al-Abdouh A., Bizanti A., Gill G., Houssien A., Alshebani Y., Kanmanthareddy A., Dahal K. (2021). Ischemic and bleeding outcomes of potent P2Y12 inhibitor antiplatelet agents versus clopidogrel in elderly patients with acute coronary syndrome: A meta-analysis of randomized trials. Cardiovasc. Revasc. Med..

[138] Pereira N.L., Rihal C., Lennon R., Marcus G., Shrivastava S., Bell M.R., So D., Geller N., Goodman S.G., Hasan A. (2021). Effect of CYP2C19 Genotype on Ischemic Outcomes During Oral P2Y12 Inhibitor Therapy: A Meta-Analysis. JACC Cardiovasc. Interv..

[139] Galli M., Benenati S., Franchi F., Rollini F., Capodanno D., Biondi-Zoccai G., Vescovo G.M., Cavallari L.H., Bikdeli B., Ten Berg J. (2021). Comparative effects of guided vs. potent P2Y12 inhibitor therapy in acute coronary syndrome: A network meta-analysis of 61 898 patients from 15 randomized trials. Eur. Heart J..

[140] Shoji S., Kuno T., Fujisaki T., Takagi H., Briasoulis A., Deharo P., Cuisset T., Latib A., Kohsaka S. (2021). De-Escalation of Dual Antiplatelet Therapy in Patients With Acute Coronary Syndromes. J. Am. Coll. Cardiol..

[141] Rey M., Kramberg M., Hess P., Morrison K., Ernst R., Haag F., Weber E., Clozel M., Baumann M., Caroff E. (2017). The reversible P2Y12 antagonist ACT-246475 causes significantly less blood loss than ticagrelor at equivalent antithrombotic efficacy in rat. Pharm. Res. Perspect..

[142] Crescence L., Darbousset R., Caroff E., Hubler F., Riederer M.A., Panicot-Dubois L., Dubois C. (2021). Selatogrel, a reversible P2Y12 receptor antagonist, has reduced off-target interference with haemostatic factors in a mouse thrombosis model. Thromb. Res..

[143] Storey R.F., Gurbel P.A., Ten Berg J., Bernaud C., Dangas G.D., Frenoux J.M., Gorog D.A., Hmissi A., Kunadian V., James S.K. (2020). Pharmacodynamics, pharmacokinetics, and safety of single-dose subcutaneous administration of selatogrel, a novel P2Y12 receptor antagonist, in patients with chronic coronary syndromes. Eur. Heart J..

[144] Sinnaeve P., Fahrni G., Schelfaut D., Spirito A., Mueller C., Frenoux J.M., Hmissi A., Bernaud C., Ufer M., Moccetti T. (2020). Subcutaneous Selatogrel Inhibits Platelet Aggregation in Patients With Acute Myocardial Infarction. J. Am. Coll. Cardiol..

[145] Schilling U., Dingemanse J., Dobrow M., Baumann M., Riederer M.A., Juif P.E., Ufer M. (2021). Insights from In Vitro and Clinical Data to Guide Transition from the Novel P2Y12 Antagonist Selatogrel to Clopidogrel, Prasugrel, and Ticagrelor. Thromb. Haemost..

[146] Crescence L., Kramberg M., Baumann M., Rey M., Roux S., Panicot-Dubois L., Dubois C., Riederer M.A. (2021). The P2Y12 Receptor Antagonist Selatogrel Dissolves Preformed Platelet Thrombi In Vivo. J. Clin. Med..

[147] Boersma E., Harrington R.A., Moliterno D.J., White H., Theroux P., Van de Werf F., de Torbal A., Armstrong P.W., Wallentin L.C., Wilcox R.G. (2002). Platelet glycoprotein IIb/IIIa inhibitors in acute coronary syndromes: A meta-analysis of all major randomised clinical trials. Lancet.

[148] Safley D.M., Venkitachalam L., Kennedy K.F., Cohen D.J. (2015). Impact of Glycoprotein IIb/IIIa Inhibition in Contemporary Percutaneous Coronary Intervention for Acute Coronary Syndromes: Insights From the National Cardiovascular Data Registry. JACC Cardiovasc. Interv..

[149] Saleiro C., Teixeira R., De Campos D., Lopes J., Oliveiros B., Costa M., Gonçalves L. (2020). Glycoprotein IIb/IIIa inhibitors for cardiogenic shock complicating acute myocardial infarction: A systematic review, meta-analysis, and meta-regression. J. Intensive Care.

[150] Ciccone A., Motto C., Abraha I., Cozzolino F., Santilli I. (2014). Glycoprotein IIb-IIIa inhibitors for acute ischaemic stroke. Cochrane Database Syst. Rev..

[151] Zhu X., Cao G. (2020). Safety of Glycoprotein IIb-IIIa Inhibitors Used in Stroke-Related Treatment: A Systematic Review and Meta-Analysis. Clin. Appl. Thromb. Hemost..

[152] Gurbel P.A., Jeong Y.H., Tantry U.S. (2011). Vorapaxar: A novel protease-activated receptor-1 inhibitor. Expert Opin. Investig. Drugs.

[153] Morrow D.A., Braunwald E., Bonaca M.P., Ameriso S.F., Dalby A.J., Fish M.P., Fox K.A., Lipka L.J., Liu X., Nicolau J.C. (2012). Vorapaxar in the secondary prevention of atherothrombotic events. N. Engl. J. Med..

[154] Tricoci P., Huang Z., Held C., Moliterno D.J., Armstrong P.W., Van de Werf F., White H.D., Aylward P.E., Wallentin L., Chen E. (2012). Thrombin-receptor antagonist vorapaxar in acute coronary syndromes. N. Engl. J. Med..

[155] Cavender M.A., Scirica B.M., Bonaca M.P., Angiolillo D.J., Dalby A.J., Dellborg M., Morais J., Murphy S.A., Ophuis T.O., Tendera M. (2015). Vorapaxar in patients with diabetes mellitus and previous myocardial infarction: Findings from the thrombin receptor antagonist in secondary prevention of atherothrombotic ischemic events-TIMI 50 trial. Circulation.

[156] Correa S., Bonaca M.P., Scirica B.M., Murphy S.A., Goodrich E.L., Morrow D.A., O’Donoghue M.L. (2019). Efficacy and safety of more potent antiplatelet therapy with vorapaxar in patients with impaired renal function. J. Thromb. Thrombolysis.

[157] Bonaca M.P., Scirica B.M., Braunwald E., Wiviott S.D., O’Donoghue M.L., Murphy S.A., Morrow D.A. (2014). Coronary stent thrombosis with vorapaxar versus placebo: Results from the TRA 2 degrees P-TIMI 50 trial. J. Am. Coll. Cardiol..

[158] Ulrichts H., Silence K., Schoolmeester A., de Jaegere P., Rossenu S., Roodt J., Priem S., Lauwereys M., Casteels P., Van Bockstaele F. (2011). Antithrombotic drug candidate ALX-0081 shows superior preclinical efficacy and safety compared with currently marketed antiplatelet drugs. Blood.

[159] Peyvandi F., Scully M., Kremer Hovinga J.A., Cataland S., Knobl P., Wu H., Artoni A., Westwood J.P., Mansouri Taleghani M., Jilma B. (2016). Caplacizumab for Acquired Thrombotic Thrombocytopenic Purpura. N. Engl. J. Med..

[160] Scully M., Cataland S.R., Peyvandi F., Coppo P., Knobl P., Kremer Hovinga J.A., Metjian A., de la Rubia J., Pavenski K., Callewaert F. (2019). Caplacizumab Treatment for Acquired Thrombotic Thrombocytopenic Purpura. N. Engl. J. Med..

[161] Peyvandi F., Scully M., Kremer Hovinga J.A., Knobl P., Cataland S., De Beuf K., Callewaert F., De Winter H., Zeldin R.K. (2017). Caplacizumab reduces the frequency of major thromboembolic events, exacerbations and death in patients with acquired thrombotic thrombocytopenic purpura. J. Thromb. Haemost..

[162] Van Loon J.E., De Jaegere P.P., Ulrichts H., Van Vliet H.H., De Maat M.P., De Groot P.G., Simoons M.L., Leebeek F.W. (2011). The in vitro effect of the new antithrombotic drug candidate ALX-0081 on blood samples of patients undergoing percutaneous coronary intervention. Thromb. Haemost..

[163] Momi S., Tantucci M., Van Roy M., Ulrichts H., Ricci G., Gresele P. (2013). Reperfusion of cerebral artery thrombosis by the GPIb-VWF blockade with the Nanobody ALX-0081 reduces brain infarct size in guinea pigs. Blood.

[164] Chen C., Kan Y., Shi Z., Guo D., Fu W., Li Y., Lv Q., Li X., Si Y. (2020). Low Dose Rivaroxaban for Atherosclerotic Cardiovascular Diseases: A Systematic Review and Meta-analysis. Front. Pharm..

[165] Oi K., Shimizu M., Natori T., Tsuda K., Yoshida M., Kamada A., Ishigaku Y., Narumi S., Oura K., Maeda T. (2021). Influence of PAR-1 in patients with non-valvular atrial fibrillation: The antiplatelet effect of dabigatran. Thromb. Res..

[166] Vinholt P.J., Nielsen C., Soderstrom A.C., Brandes A., Nybo M. (2017). Dabigatran reduces thrombin-induced platelet aggregation and activation in a dose-dependent manner. J. Thromb. Thrombolysis.

[167] Kim J., Jang H.J., Schellingerhout D., Lee S.K., Kim H., Kim Y.D., Lee K.Y., Choi H.Y., Cho H.J., Jang S.S. (2021). Short-Term Cessation of Dabigatran Causes a Paradoxical Prothrombotic State. Ann. Neurol..

[168] Polzin A., Dannenberg L., Thienel M., Orban M., Wolff G., Hohlfeld T., Zeus T., Kelm M., Petzold T. (2021). Noncanonical Effects of Oral Thrombin and Factor Xa Inhibitors in Platelet Activation and Arterial Thrombosis. Thromb. Haemost..

[169] Arantes F.B.B., Menezes F.R., Franci A., Barbosa C., Dalcoquio T.F., Nakashima C.A.K., Baracioli L.M., Furtado R.H.M., Nomelini Q.S.S., Ramires J.A.F. (2020). Influence of Direct Thrombin Inhibitor and Low Molecular Weight Heparin on Platelet Function in Patients with Coronary Artery Disease: A Prospective Interventional Trial. Adv. Ther..

[170] Jourdi G., Bachelot-Loza C., Mazoyer E., Poirault-Chassac S., Duchemin J., Fontenay M., Gaussem P. (2019). Effect of rivaroxaban and dabigatran on platelet functions: In vitro study. Thromb. Res..

[171] Shah B., Allen N., Harchandani B., Pillinger M., Katz S., Sedlis S.P., Echagarruga C., Samuels S.K., Morina P., Singh P. (2016). Effect of Colchicine on Platelet-Platelet and Platelet-Leukocyte Interactions: A Pilot Study in Healthy Subjects. Inflammation.

[172] Cirillo P., Taglialatela V., Pellegrino G., Morello A., Conte S., Di Serafino L., Cimmino G. (2020). Effects of colchicine on platelet aggregation in patients on dual antiplatelet therapy with aspirin and clopidogrel. J. Thromb. Thrombolysis.

[173] Cimmino G., Tarallo R., Conte S., Morello A., Pellegrino G., Loffredo F.S., Cali G., De Luca N., Golino P., Trimarco B. (2018). Colchicine reduces platelet aggregation by modulating cytoskeleton rearrangement via inhibition of cofilin and LIM domain kinase 1. Vasc. Pharm..

[174] Pennings G.J., Reddel C.J., Traini M., Campbell H., Chen V., Kritharides L. (2021). Colchicine inhibits ROS generation in response to glycoprotein VI stimulation. Sci. Rep..

[175] Fiolet A.T.L., Opstal T.S.J., Mosterd A., Eikelboom J.W., Jolly S.S., Keech A.C., Kelly P., Tong D.C., Layland J., Nidorf S.M. (2021). Efficacy and safety of low-dose colchicine in patients with coronary disease: A systematic review and meta-analysis of randomized trials. Eur. Heart J..

[176] Manfredi A.A., Baldini M., Camera M., Baldissera E., Brambilla M., Peretti G., Maseri A., Rovere-Querini P., Tremoli E., Sabbadini M.G. (2016). Anti-TNFalpha agents curb platelet activation in patients with rheumatoid arthritis. Ann. Rheum. Dis..

[177] Padfield G.J., Din J.N., Koushiappi E., Mills N.L., Robinson S.D., Cruden Nle M., Lucking A.J., Chia S., Harding S.A., Newby D.E. (2013). Cardiovascular effects of tumour necrosis factor alpha antagonism in patients with acute myocardial infarction: A first in human study. Heart.

[178] Nielsen C.B., Nielsen C., Nybo M., Just S.A., Vinholt P.J. (2020). The in vitro effect of antirheumatic drugs on platelet function. Platelets.

[179] Canzano P., Brambilla M., Porro B., Cosentino N., Tortorici E., Vicini S., Poggio P., Cascella A., Pengo M.F., Veglia F. (2021). Platelet and Endothelial Activation as Potential Mechanisms Behind the Thrombotic Complications of COVID-19 Patients. JACC Basic Transl. Sci..

[180] Ridker P.M., Everett B.M., Thuren T., MacFadyen J.G., Chang W.H., Ballantyne C., Fonseca F., Nicolau J., Koenig W., Anker S.D. (2017). Antiinflammatory Therapy with Canakinumab for Atherosclerotic Disease. N. Engl. J. Med..

[181] DeSena A.D., Do T., Schulert G.S. (2018). Systemic autoinflammation with intractable epilepsy managed with interleukin-1 blockade. J. Neuroinflamm..

[182] Dobie G., Kuriri F.A., Omar M.M.A., Alanazi F., Gazwani A.M., Tang C.P.S., Sze D.M., Handunnetti S.M., Tam C., Jackson D.E. (2019). Ibrutinib, but not zanubrutinib, induces platelet receptor shedding of GPIb-IX-V complex and integrin alphaIIbbeta3 in mice and humans. Blood Adv..

[183] Ninomoto J., Mokatrin A., Kinoshita T., Marimpietri C., Barrett T.D., Chang B.Y., Sukbuntherng J., James D.F., Crowther M. (2020). Effects of ibrutinib on in vitro platelet aggregation in blood samples from healthy donors and donors with platelet dysfunction. Hematology.

[184] Nicolson P.L.R., Nock S.H., Hinds J., Garcia-Quintanilla L., Smith C.W., Campos J., Brill A., Pike J.A., Khan A.O., Poulter N.S. (2021). Low-dose Btk inhibitors selectively block platelet activation by CLEC-2. Haematologica.

[185] Diener J.L., Daniel Lagasse H.A., Duerschmied D., Merhi Y., Tanguay J.F., Hutabarat R., Gilbert J., Wagner D.D., Schaub R. (2009). Inhibition of von Willebrand factor-mediated platelet activation and thrombosis by the anti-von Willebrand factor A1-domain aptamer ARC1779. J. Thromb. Haemost..

[186] Spiel A.O., Mayr F.B., Ladani N., Wagner P.G., Schaub R.G., Gilbert J.C., Jilma B. (2009). The aptamer ARC1779 is a potent and specific inhibitor of von Willebrand Factor mediated ex vivo platelet function in acute myocardial infarction. Platelets.

[187] Gilbert J.C., DeFeo-Fraulini T., Hutabarat R.M., Horvath C.J., Merlino P.G., Marsh H.N., Healy J.M., Boufakhreddine S., Holohan T.V., Schaub R.G. (2007). First-in-human evaluation of anti von Willebrand factor therapeutic aptamer ARC1779 in healthy volunteers. Circulation.

[188] Markus H.S., McCollum C., Imray C., Goulder M.A., Gilbert J., King A. (2011). The von Willebrand inhibitor ARC1779 reduces cerebral embolization after carotid endarterectomy: A randomized trial. Stroke.

[189] Sakai K., Someya T., Harada K., Yagi H., Matsui T., Matsumoto M. (2020). Novel aptamer to von Willebrand factor A1 domain (TAGX-0004) shows total inhibition of thrombus formation superior to ARC1779 and comparable to caplacizumab. Haematologica.

[190] Kovacevic K.D., Buchtele N., Schoergenhofer C., Derhaschnig U., Gelbenegger G., Brostjan C., Zhu S., Gilbert J.C., Jilma B. (2020). The aptamer BT200 effectively inhibits von Willebrand factor (VWF) dependent platelet function after stimulated VWF release by desmopressin or endotoxin. Sci. Rep..

[191] Kovacevic K.D., Jilma B., Zhu S., Gilbert J.C., Winter M.P., Toma A., Hengstenberg C., Lang I., Kubica J., Siller-Matula J.M. (2020). von Willebrand Factor Predicts Mortality in ACS Patients Treated with Potent P2Y12 Antagonists and is Inhibited by Aptamer BT200 Ex Vivo. Thromb. Haemost..

[192] Kovacevic K.D., Greisenegger S., Langer A., Gelbenegger G., Buchtele N., Pabinger I., Petroczi K., Zhu S., Gilbert J.C., Jilma B. (2021). The aptamer BT200 blocks von Willebrand factor and platelet function in blood of stroke patients. Sci. Rep..

[193] Kovacevic K.D., Grafeneder J., Schorgenhofer C., Gelbenegger G., Gager G., Firbas C., Quehenberger P., Jilma-Stohlawetz P., Bileck A., Zhu S. (2021). The von Willebrand Factor A-1 domain binding aptamer BT200 elevates plasma levels of VWF and Factor VIII: A first-in-human trial. Haematologica.

[194] Nimjee S.M., Dornbos D., Pitoc G.A., Wheeler D.G., Layzer J.M., Venetos N., Huttinger A., Talentino S.E., Musgrave N.J., Moody H. (2019). Preclinical Development of a vWF Aptamer to Limit Thrombosis and Engender Arterial Recanalization of Occluded Vessels. Mol. Ther..

[195] Lei X., Reheman A., Hou Y., Zhou H., Wang Y., Marshall A.H., Liang C., Dai X., Li B.X., Vanhoorelbeke K. (2014). Anfibatide, a novel GPIb complex antagonist, inhibits platelet adhesion and thrombus formation in vitro and in vivo in murine models of thrombosis. Thromb. Haemost..

[196] Li T.T., Fan M.L., Hou S.X., Li X.Y., Barry D.M., Jin H., Luo S.Y., Kong F., Lau L.F., Dai X.R. (2015). A novel snake venom-derived GPIb antagonist, anfibatide, protects mice from acute experimental ischaemic stroke and reperfusion injury. Br. J. Pharm..

[197] Chu W., Sun X., Zhu X., Zhao Y.C., Zhang J., Kong Q., Zhou L. (2021). Blockade of platelet glycoprotein receptor Ib ameliorates blood-brain barrier disruption following ischemic s.stroke via Epac pathway. Biomed. Pharm..

[198] Li B.X., Dai X., Xu X.R., Adili R., Neves M.A.D., Lei X., Shen C., Zhu G., Wang Y., Zhou H. (2021). In vitro assessment and phase I randomized clinical trial of anfibatide a snake venom derived anti-thrombotic agent targeting human platelet GPIbalpha. Sci. Rep..

[199] Zheng B., Li J., Jiang J., Xiang D., Chen Y., Yu Z., Zeng H., Ge J., Dai X., Liu J. (2021). Safety and efficacy of a platelet glycoprotein Ib inhibitor for patients with non-ST segment elevation myocardial infarction: A phase Ib/IIa study. Pharmacotherapy.

[200] Fontayne A., Meiring M., Lamprecht S., Roodt J., Demarsin E., Barbeaux P., Deckmyn H. (2008). The humanized anti-glycoprotein Ib monoclonal antibody h6B4-Fab is a potent and safe antithrombotic in a high shear arterial thrombosis model in baboons. Thromb. Haemost..

[201] Schulz C., Penz S., Hoffmann C., Langer H., Gillitzer A., Schneider S., Brandl R., Seidl S., Massberg S., Pichler B. (2008). Platelet GPVI binds to collagenous structures in the core region of human atheromatous plaque and is critical for atheroprogression in vivo. Basic Res. Cardiol..

[202] Massberg S., Konrad I., Bultmann A., Schulz C., Munch G., Peluso M., Lorenz M., Schneider S., Besta F., Muller I. (2004). Soluble glycoprotein VI dimer inhibits platelet adhesion and aggregation to the injured vessel wall in vivo. FASEB J..

[203] Goebel S., Li Z., Vogelmann J., Holthoff H.P., Degen H., Hermann D.M., Gawaz M., Ungerer M., Munch G. (2013). The GPVI-Fc fusion protein Revacept improves cerebral infarct volume and functional outcome in stroke. PLoS ONE.

[204] Mojica Munoz A.K., Jamasbi J., Uhland K., Degen H., Munch G., Ungerer M., Brandl R., Megens R., Weber C., Lorenz R. (2017). Recombinant GPVI-Fc added to single or dual antiplatelet therapy in vitro prevents plaque-induced platelet thrombus formation. Thromb. Haemost..

[205] Degen H., Borst O., Ziegler M., Mojica Munoz A.K., Jamasbi J., Walker B., Gobel S., Fassbender J., Adler K., Brandl R. (2017). ADPase CD39 Fused to Glycoprotein VI-Fc Boosts Local Antithrombotic Effects at Vascular Lesions. J. Am. Heart Assoc..

[206] Mayer K., Hein-Rothweiler R., Schupke S., Janisch M., Bernlochner I., Ndrepepa G., Sibbing D., Gori T., Borst O., Holdenrieder S. (2021). Efficacy and Safety of Revacept, a Novel Lesion-Directed Competitive Antagonist to Platelet Glycoprotein VI, in Patients Undergoing Elective Percutaneous Coronary Intervention for Stable Ischemic Heart Disease: The Randomized, Double-blind, Placebo-Controlled ISAR-PLASTER Phase 2 Trial. JAMA Cardiol..

[207] Groschel K., Uphaus T., Loftus I., Poppert H., Diener H.C., Zobel J., Munch G. (2020). Revacept, an Inhibitor of Platelet Adhesion in Symptomatic Carotid Artery Stenosis: Design and Rationale of a Randomized Phase II Clinical Trial. TH Open.

[208] Lebozec K., Jandrot-Perrus M., Avenard G., Favre-Bulle O., Billiald P. (2017). Design, development and characterization of ACT017, a humanized Fab that blocks platelet’s glycoprotein VI function without causing bleeding risks. MAbs.

[209] Renaud L., Lebozec K., Voors-Pette C., Dogterom P., Billiald P., Jandrot Perrus M., Pletan Y., Machacek M. (2020). Population Pharmacokinetic/Pharmacodynamic Modeling of Glenzocimab (ACT017) a Glycoprotein VI Inhibitor of Collagen-Induced Platelet Aggregation. J. Clin. Pharm..

[210] Ahmed M.U., Kaneva V., Loyau S., Nechipurenko D., Receveur N., Le Bris M., Janus-Bell E., Didelot M., Rauch A., Susen S. (2020). Pharmacological Blockade of Glycoprotein VI Promotes Thrombus Disaggregation in the Absence of Thrombin. Arter. Thromb. Vasc. Biol..

[211] Tsukiji N., Osada M., Sasaki T., Shirai T., Satoh K., Inoue O., Umetani N., Mochizuki C., Saito T., Kojima S. (2018). Cobalt hematoporphyrin inhibits CLEC-2-podoplanin interaction, tumor metastasis, and arterial/venous thrombosis in mice. Blood Adv..

[212] Chang Y.W., Hsieh P.W., Chang Y.T., Lu M.H., Huang T.F., Chong K.Y., Liao H.R., Cheng J.C., Tseng C.P. (2015). Identification of a novel platelet antagonist that binds to CLEC-2 and suppresses podoplanin-induced platelet aggregation and cancer metastasis. Oncotarget.

[213] Carminita E., Crescence L., Brouilly N., Altie A., Panicot-Dubois L., Dubois C. (2021). DNAse-dependent, NET-independent pathway of thrombus formation in vivo. Proc. Natl. Acad. Sci. USA.

[214] Li T., Wang C., Liu Y., Li B., Zhang W., Wang L., Yu M., Zhao X., Du J., Zhang J. (2020). Neutrophil Extracellular Traps Induce Intestinal Damage and Thrombotic Tendency in Inflammatory Bowel Disease. J. Crohns Colitis.

[215] Novotny J., Chandraratne S., Weinberger T., Philippi V., Stark K., Ehrlich A., Pircher J., Konrad I., Oberdieck P., Titova A. (2018). Histological comparison of arterial thrombi in mice and men and the influence of Cl-amidine on thrombus formation. PLoS ONE.

[216] Clark S.R., Ma A.C., Tavener S.A., McDonald B., Goodarzi Z., Kelly M.M., Patel K.D., Chakrabarti S., McAvoy E., Sinclair G.D. (2007). Platelet TLR4 activates neutrophil extracellular traps to ensnare bacteria in septic blood. Nat. Med..

[217] Sun J., Zhang M., Chen K., Chen B., Zhao Y., Gong H., Zhao X., Qi R. (2018). Suppression of TLR4 activation by resveratrol is associated with STAT3 and Akt inhibition in oxidized low-density lipoprotein-activated platelets. Eur. J. Pharm..

[218] Huang W.C., Liu J.C., Hsia C.W., Fong T.H., Hsia C.H., Tran O.T., Velusamy M., Yang C.H., Sheu J.R. (2021). Pterostilbene, a Dimethylether Analogue of Resveratrol, Possesses High Potency in the Prevention of Platelet Activation in Humans and the Reduction of Vascular Thrombosis in Mice. J. Agric. Food Chem..

[219] Cornelius D.C., Travis O.K., Tramel R.W., Borges-Rodriguez M., Baik C.H., Greer M., Giachelli C.A., Tardo G.A., Williams J.M. (2020). NLRP3 inflammasome inhibition attenuates sepsis-induced platelet activation and prevents multi-organ injury in cecal-ligation puncture. PLoS ONE.

[220] Wang Y., Jiang H., Hu X., Fu W. (2021). Bone marrow NLRP3 inflammasome-IL-1beta signal regulates post-myocardial infarction megakaryocyte development and platelet production. Biochem. Biophys. Res. Commun..

[221] Garcia A., Dunoyer-Geindre S., Nolli S., Strassel C., Reny J.L., Fontana P. (2021). miR-204-5p and Platelet Function Regulation: Insight into a Mechanism Mediated by CDC42 and GPIIbIIIa. Thromb. Haemost..

